# Supplementary material for: Pharmacological or genetic inhibition of iNOS prevents cachexia‐mediated muscle wasting and its associated metabolism defects
Source: EMBO Mol Med. 2021 Jun 7;13(7):e13591. doi: 10.15252/emmm.202013591 (PMC8261493; doi:10.15252/emmm.202013591)
Supplement: Supplementary file 1 — Appendix [file EMMM-13-e13591-s003.pdf]

Appendix Sadek et al. (EMM-2020-13591)

Appendix Figure S1 to S14 with figure legends

Appendix Table S1 to S19

Appendix Figures

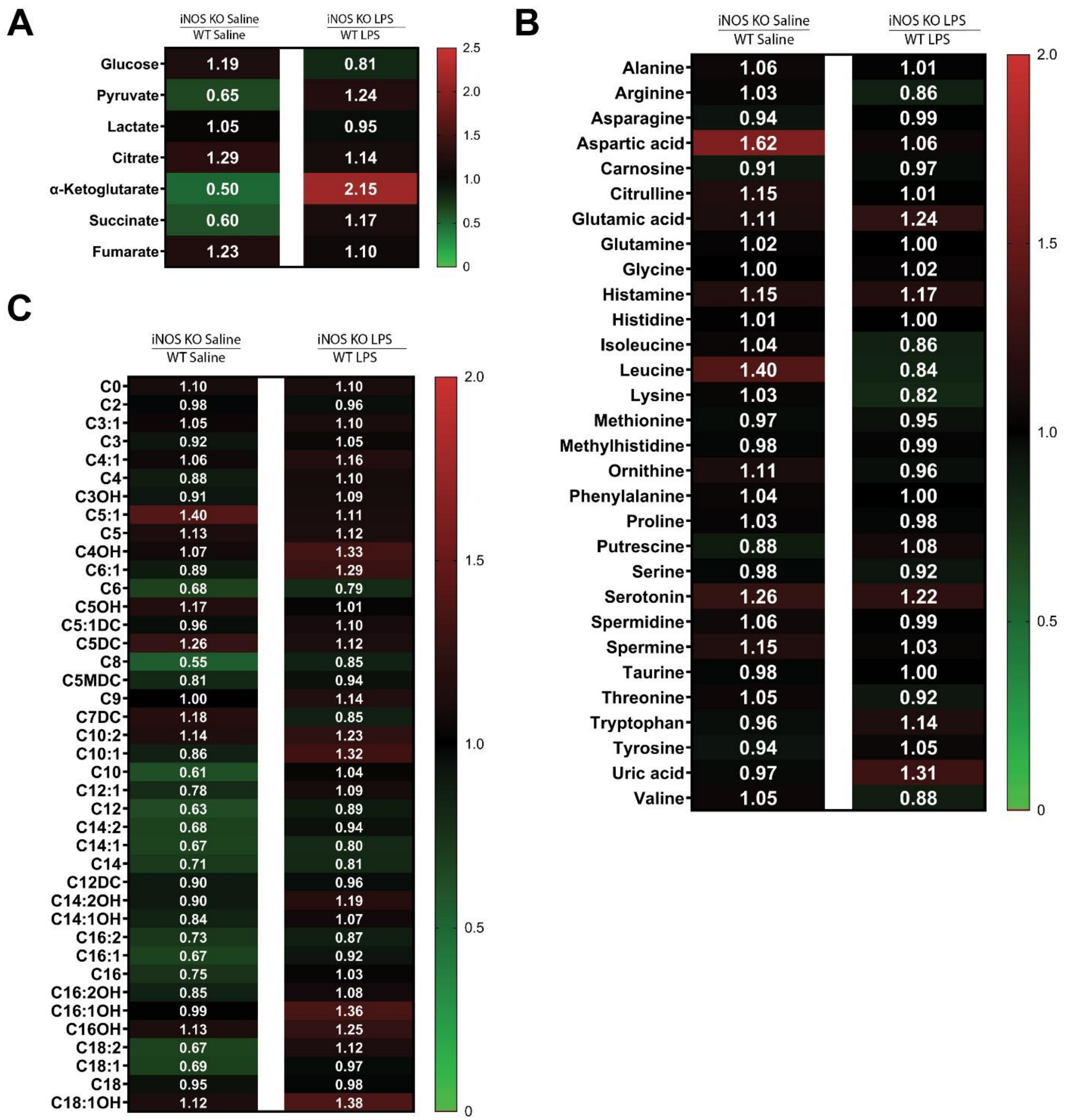

Appendix Figure S1: Metabolite levels of iNOS KO mice compared to WT mice.

Male C57BL/6 wildtype (WT) and iNOS knockout (KO) mice were intraperitoneally injected with 1mg kg^{-1} LPS or an equivalent volume of carrier solution. Saline treated WT and iNOS KO mice as well as the LPS-treated KO cohorts were pair-fed (PF) to the WT LPS-treated cohorts. After 18h, mice were euthanized and the metabolome of tibialis anterior muscles was analyzed through LC-MS/MS.

(A-C)Heatmap visualizing mean concentration of (A) metabolites involved in

Glycolysis and TCA cycle (B) amino acids and amino acid derivatives and (C) acylcarnitines relative to WT of each treatment group. Red and green indicate an increase or decrease in metabolite levels, respectively.

Data information: Individual data points represent values from individual mice, with a total of six mice per cohort (n = 6). Plotted concentration data was relativized to WT of corresponding treatment.

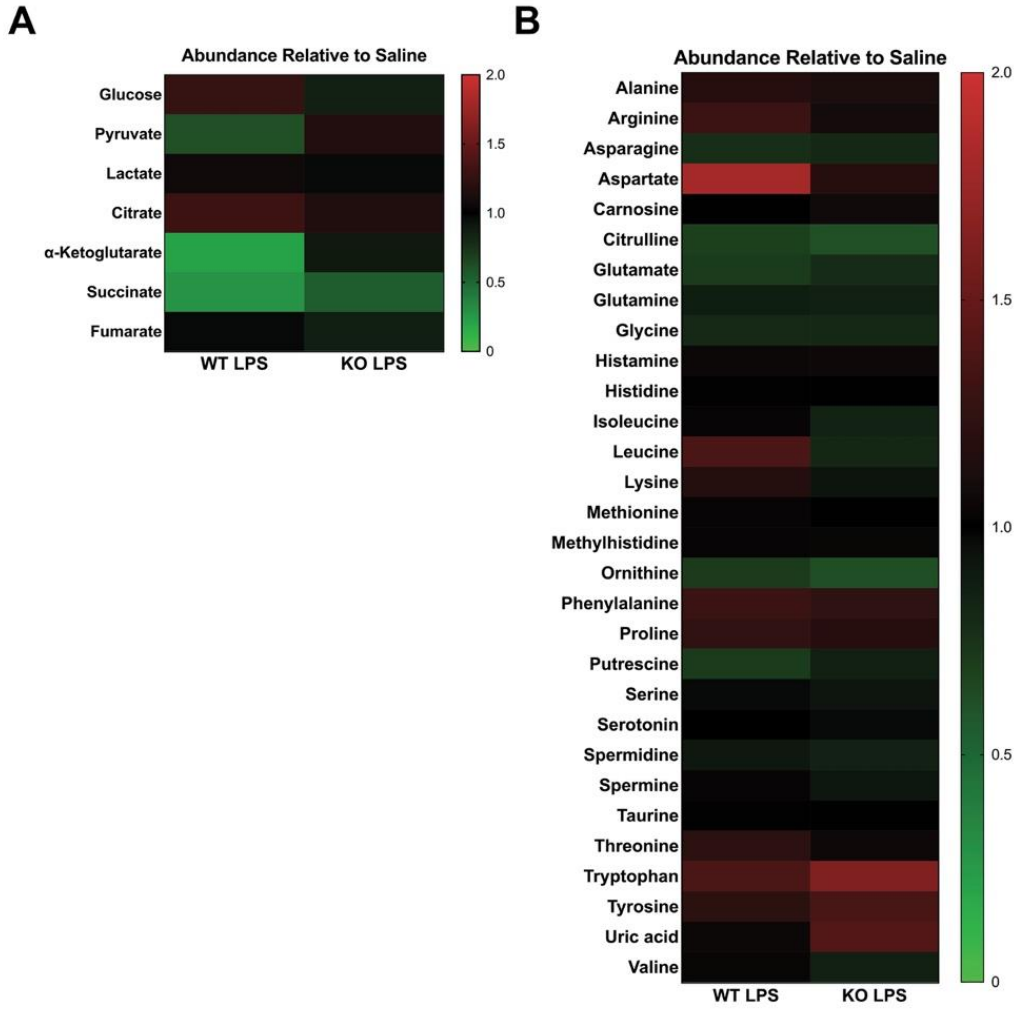

Appendix Figure S2: Genetic ablation of iNOS prevents LPS-driven deregulation of the TCA cycle and amino acid metabolism.

Male C57BL/6 wildtype (WT) and iNOS knockout (KO) mice were intraperitoneally injected with 1mg kg^{-1} LPS or an equivalent volume of carrier solution. Control WT, Control KO, and LPS-treated KO cohorts were pair-fed (PF) to the WT LPS-treated cohorts. After 18h, mice were euthanized and the metabolome of tibialis anterior muscles was analyzed through LC-MS/MS.

(A) Heatmap visualizing mean concentration corresponding to metabolites involved in Glycolysis and TCA cycle relative to saline controls of each genotype. Red and green indicate an increase or decrease in metabolite levels, respectively.
(B) Heatmap visualizing mean concentration of amino acids and amino acid derivatives relative to saline controls of each genotype. Red and green indicate an increase or decrease in metabolite levels, respectively.

Data information: Individual data points represent values from individual mice, with a total of six mice per cohort (n = 6). Plotted concentration data was relativized to saline controls of corresponding genotype.

A

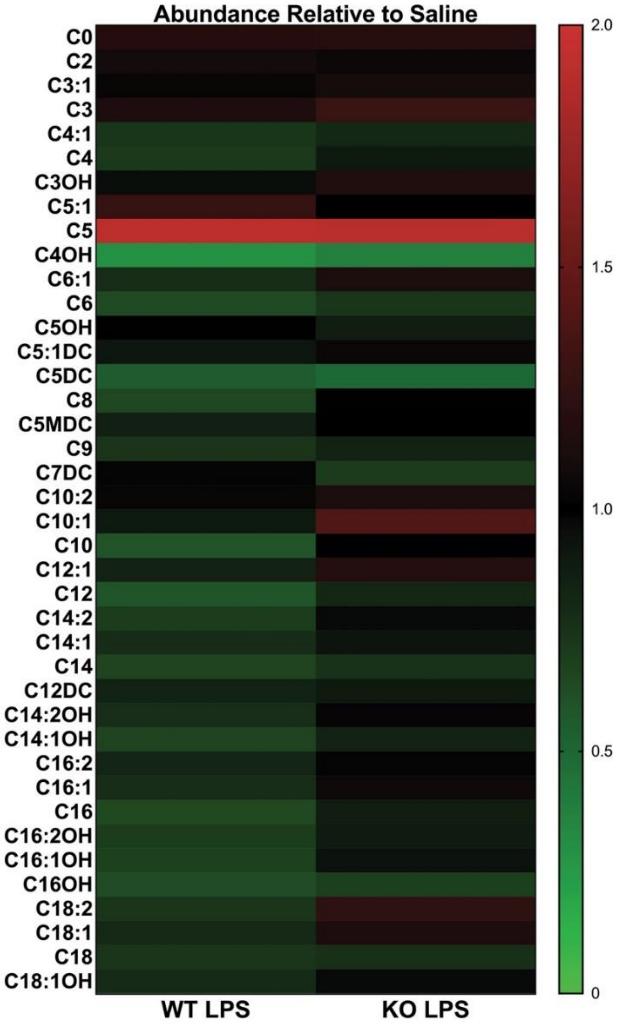

Appendix Figure S3: Genetic ablation of iNOS prevents LPS-driven deregulation of the acylcarnitine metabolism.

Male C57BL/6 wildtype (WT) and iNOS knockout (KO) mice were intraperitoneally injected with 1mg kg^{-1} LPS or an equivalent volume of carrier solution. Control WT, Control KO, and LPS-treated KO cohorts were pair-fed (PF) to the WT LPS-treated cohorts. After 18h, mice were euthanized and the metabolome of tibialis anterior muscles was analyzed through LC-MS/MS.

(A) Heatmap visualizing mean concentration of acylcarnitines relative to saline controls of each genotype. Red and green indicate an increase or decrease in metabolite levels, respectively.

Data information: Individual data points represent values from individual mice, with a total of six mice per cohort (n = 6). Plotted concentration data was relativized to saline controls of corresponding genotype.

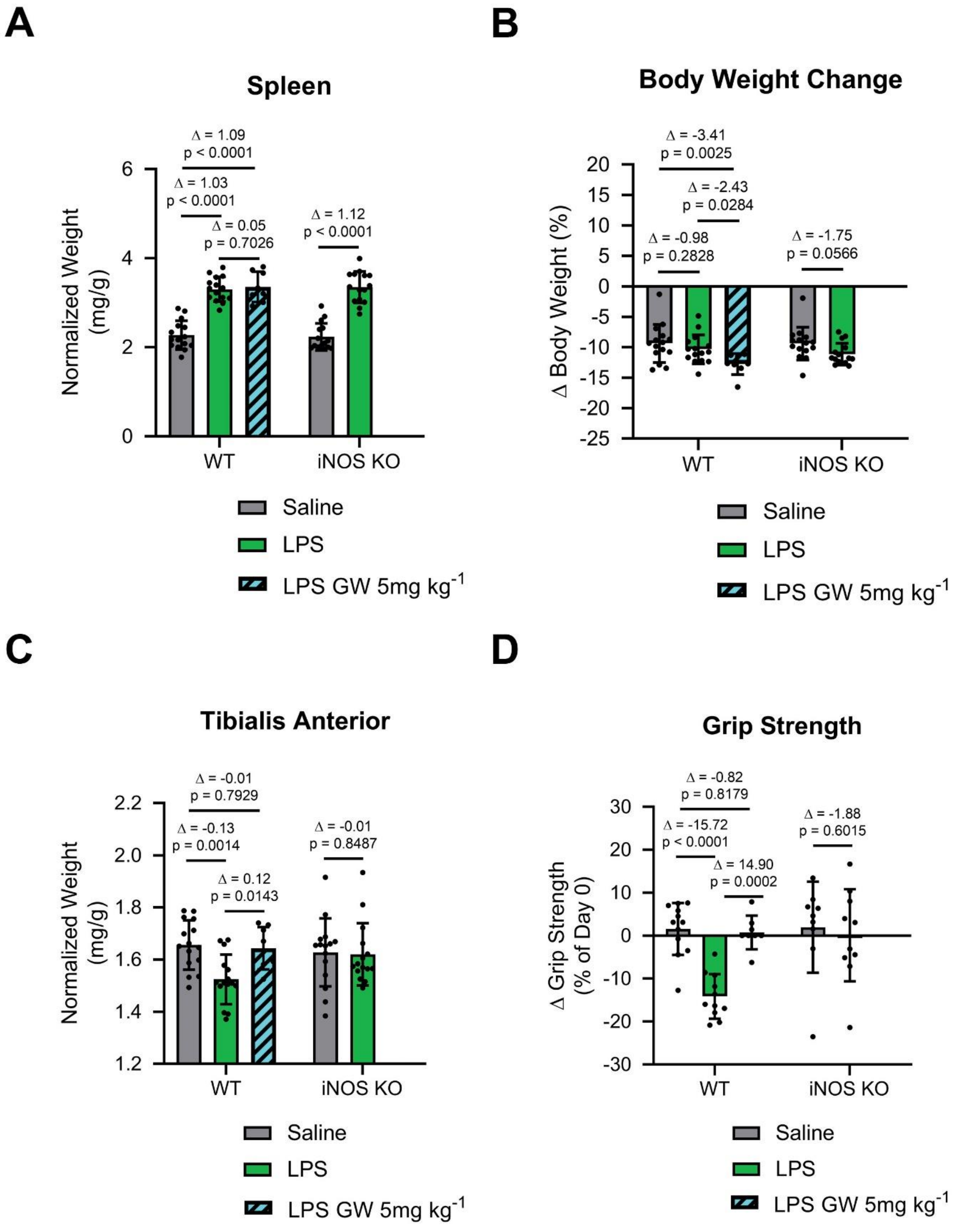

Appendix Figure S4: Pharmacological inhibition of iNOS prevents LPS-driven muscle wasting.

Male C57BL/6 wildtype (WT) and iNOS knockout (KO) mice were intraperitoneally injected with saline, 1mg kg^{-1} LPS or 5mg kg^{-1} of GW and LPS. Data was compared to previously

shown data in Figures 1D, 1H, EV1A, and EV2A from Control WT, Control KO, and LPS-treated WT and KO cohorts. After 18h, mice were euthanized, and tissue samples were analyzed.

(A) Spleen weight normalized to initial body weight. (WT saline n=15, WT LPS n=15, WT LPS+GW n=8, iNOS KO saline n=15, and iNOS KO LPS n=15).
(B) Percent body weight change from time of injection to endpoint of experiment. (WT saline n=15, WT LPS n=15, WT LPS+GW n=8, iNOS KO saline n=15, and iNOS KO LPS n=15).
(C) Tibialis anterior weight normalized to initial body weight. (WT saline n=15, WT LPS n=15, WT LPS+GW n=8, iNOS KO saline n=15, and iNOS KO LPS n=15).
(D) Change in grip strength from before injection and before endpoint collection. (WT saline n=12, WT LPS n=11, WT LPS+GW n=8, iNOS KO saline n=9, and iNOS KO LPS n=10).

Data information: Data from Control WT, Control KO, and LPS-treated WT and KO cohorts are those shown in Figures 1D, 1H, EV1A, and EV2A and compared to WT LPS and GW-treated mice. Individual data points represent values from individual mice. Error bars represent the standard deviation (SD) of the mean. For statistical comparisons, Δ indicates the difference of mean values and p-values were calculated with an ANOVA followed by Fisher's LSD test.

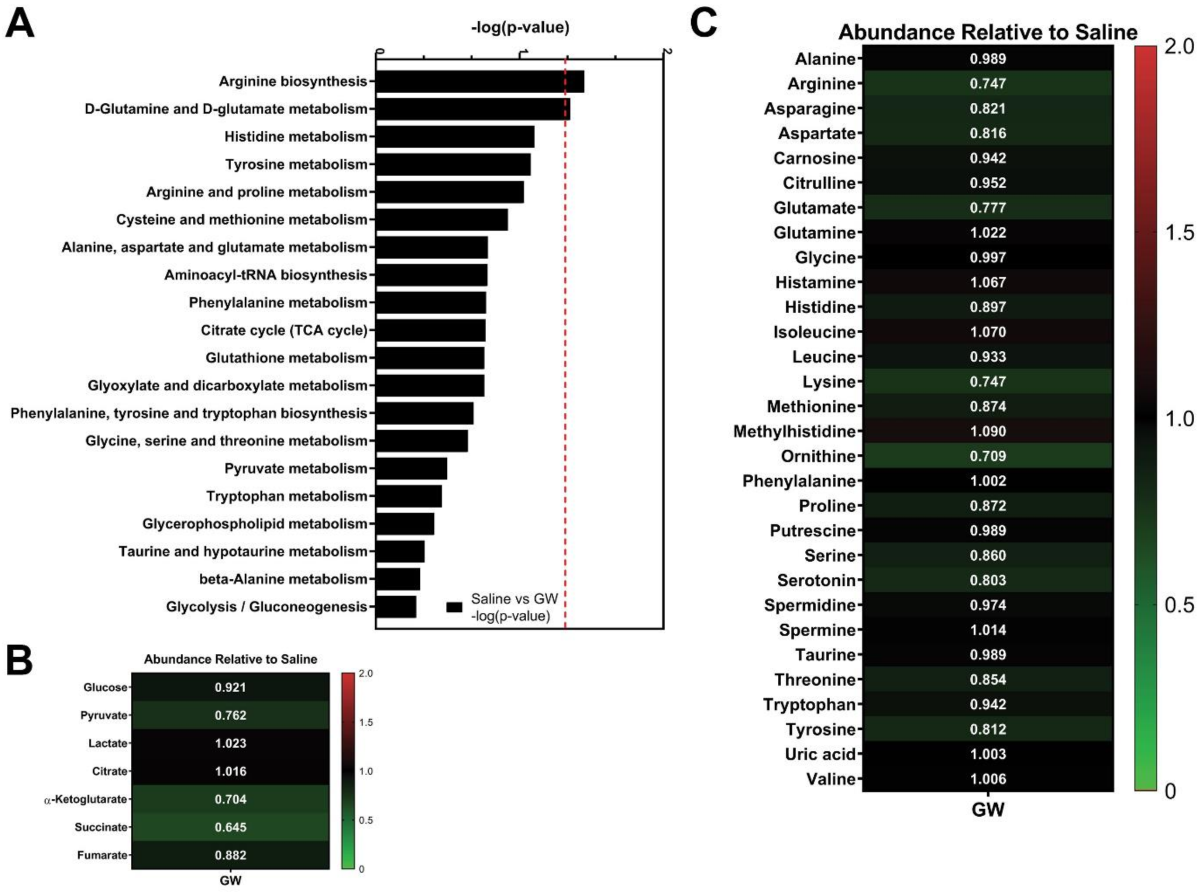

Appendix Figure S5: Characterization of GW-induced metabolome alterations.

Male BALB/C mice were injected subcutaneously with saline. After 5 days and everyday thereafter, the mice were injected with either saline or GW 5mg kg^{-1}. After 16 days, mice were euthanized and the metabolome of tibialis anterior muscles was analyzed through LC-MS/MS.

(A) Pathway Analysis using MetaboAnalyst 4.0 Software comparing significantly altered pathways from saline to GW. Pathways are ranked by their significance and filtered based on a Pathway Impact Score >0.1. Metabolomic data were range-scaled and mean-centered.

(B-C) Heatmap visualizing mean concentration corresponding to (B) metabolites involved in Glycolysis and TCA cycle or (C) amino acids and amino acid derivatives in GW treated mice relative to saline controls. Red and green indicate an increase or decrease in metabolite levels, respectively.

Data information: Individual data points represent values from individual mice, with a total of six mice per cohort (n = 6).

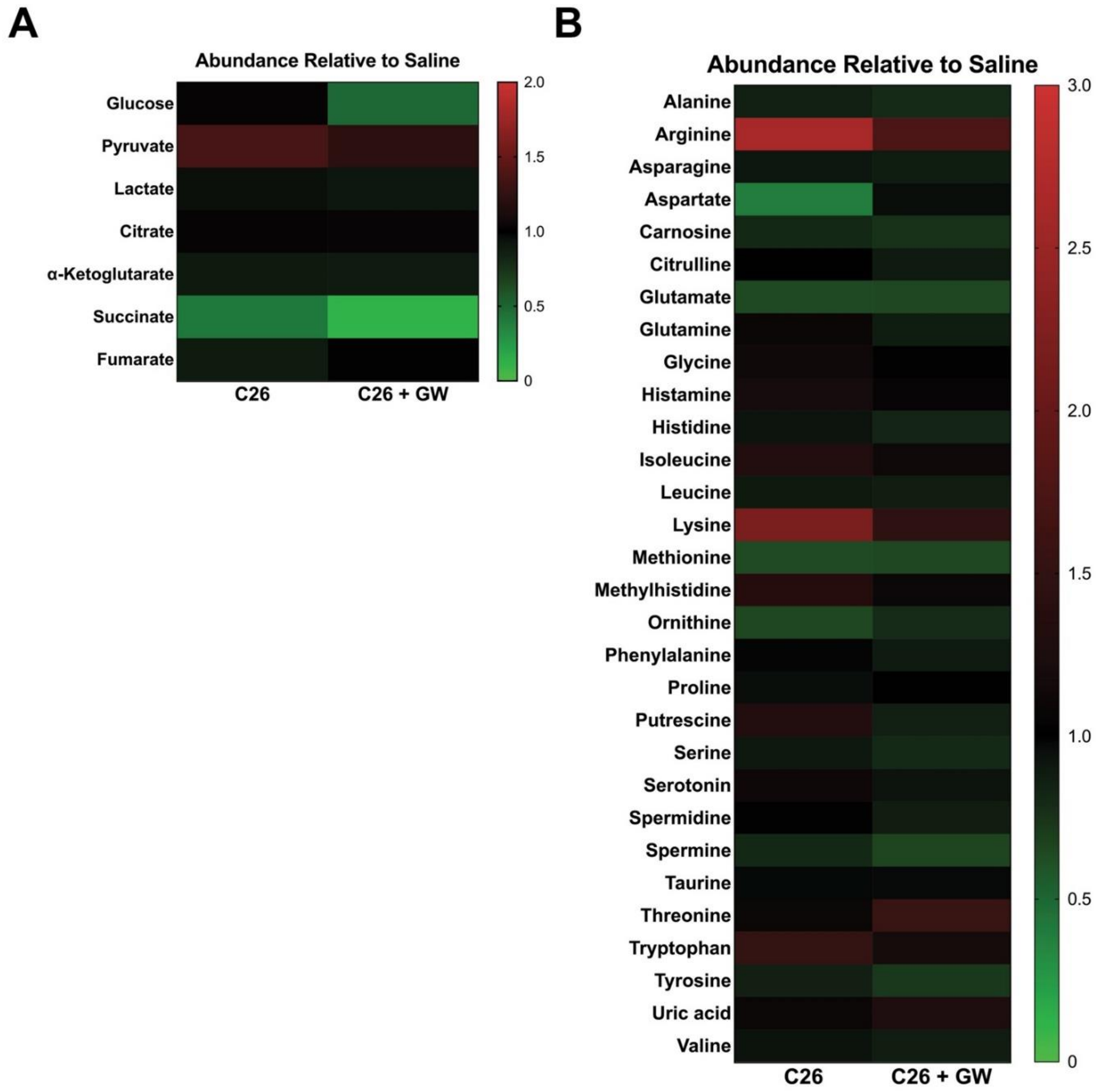

Appendix Figure S6: Pharmacological inhibition of iNOS prevents C26-driven deregulation of amino acid metabolism.

Male BALB/C mice were injected subcutaneously with C26 cells (1.25×10^6 cells) or an equivalent volume of saline. After 5 days and everyday thereafter, the mice injected with C26 cells were injected with either saline or GW 5mg kg^{-1}. After 16 days, mice were euthanized and the metabolome of tibialis anterior muscles was analyzed through LC-MS/MS.

(A-B) Heatmap visualizing mean concentration corresponding to (A) metabolites involved in Glycolysis and TCA cycle or (B) amino acids and amino acid derivatives relative to saline controls. Red and green indicate an increase or decrease in metabolite levels, respectively.

Data information: Individual data points represent values from individual mice, with a total of six mice per cohort (n = 6).

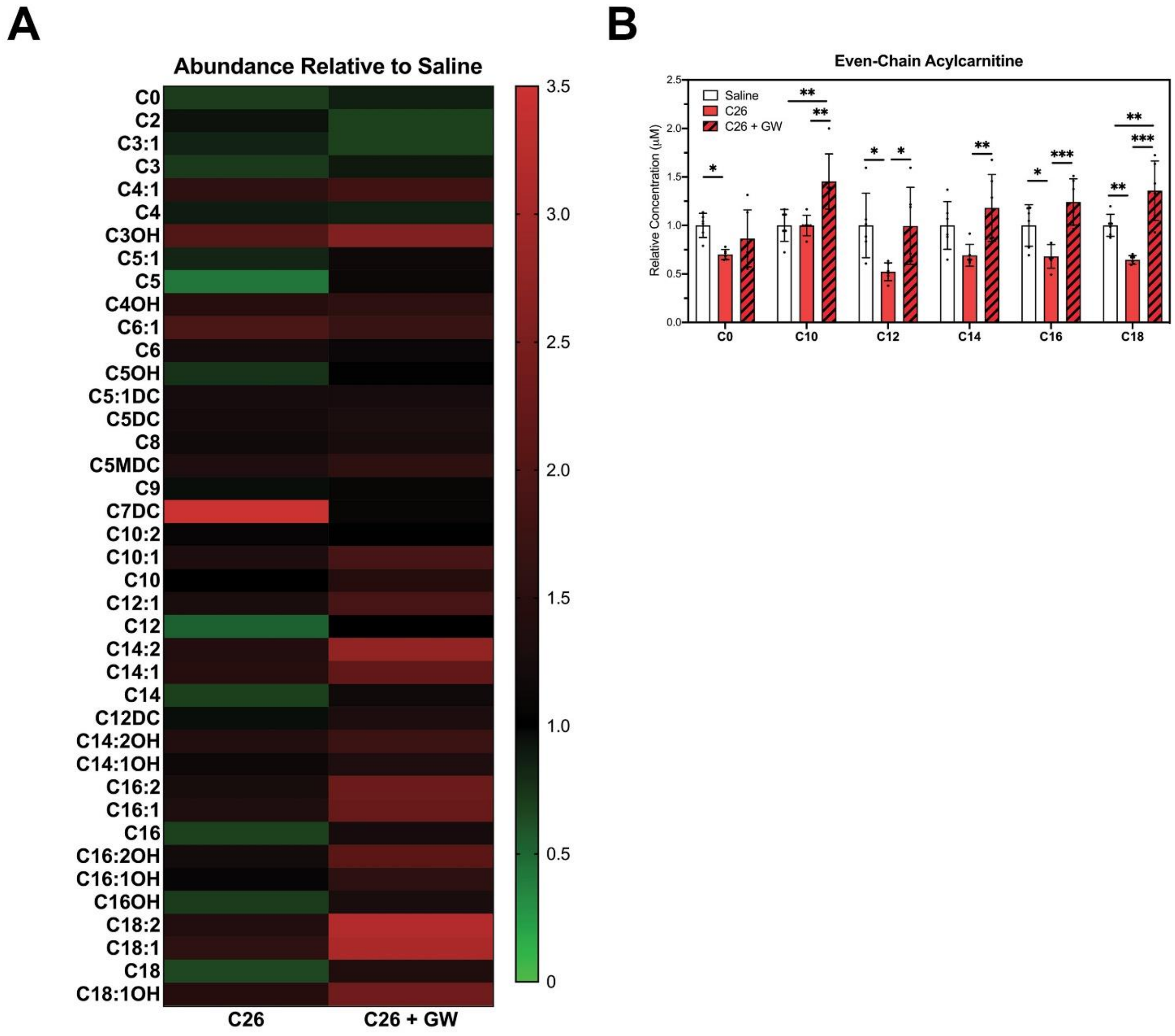

Appendix Figure S7: Pharmacological inhibition of iNOS prevents C26-driven deregulation of acylcarnitine metabolism.

Male BALB/C mice were injected subcutaneously with C26 cells ($1.25x10^{6}$ cells) or an equivalent volume of saline. After 5 days and everyday thereafter, the mice injected with C26 cells were injected with either saline or GW (5mg kg^{-1}). After 16 days, mice were euthanized and the metabolome of tibialis anterior muscles was analyzed through LC-MS/MS.

(A) Heatmap visualizing mean concentration of acylcarnitines relative to saline controls. Red and green indicate an increase or decrease in metabolite levels, respectively.
(B) Relative concentrations of even-chain acylcarnitines.

Data information: Individual data points represent values from individual mice, with a total of six mice per cohort (n = 6). Error bars represent the standard deviation (SD) of the mean. p-values were calculated with an ANOVA followed by Fisher's LSD test (*P < 0.05; **P < 0.01; ***P < 0.001).

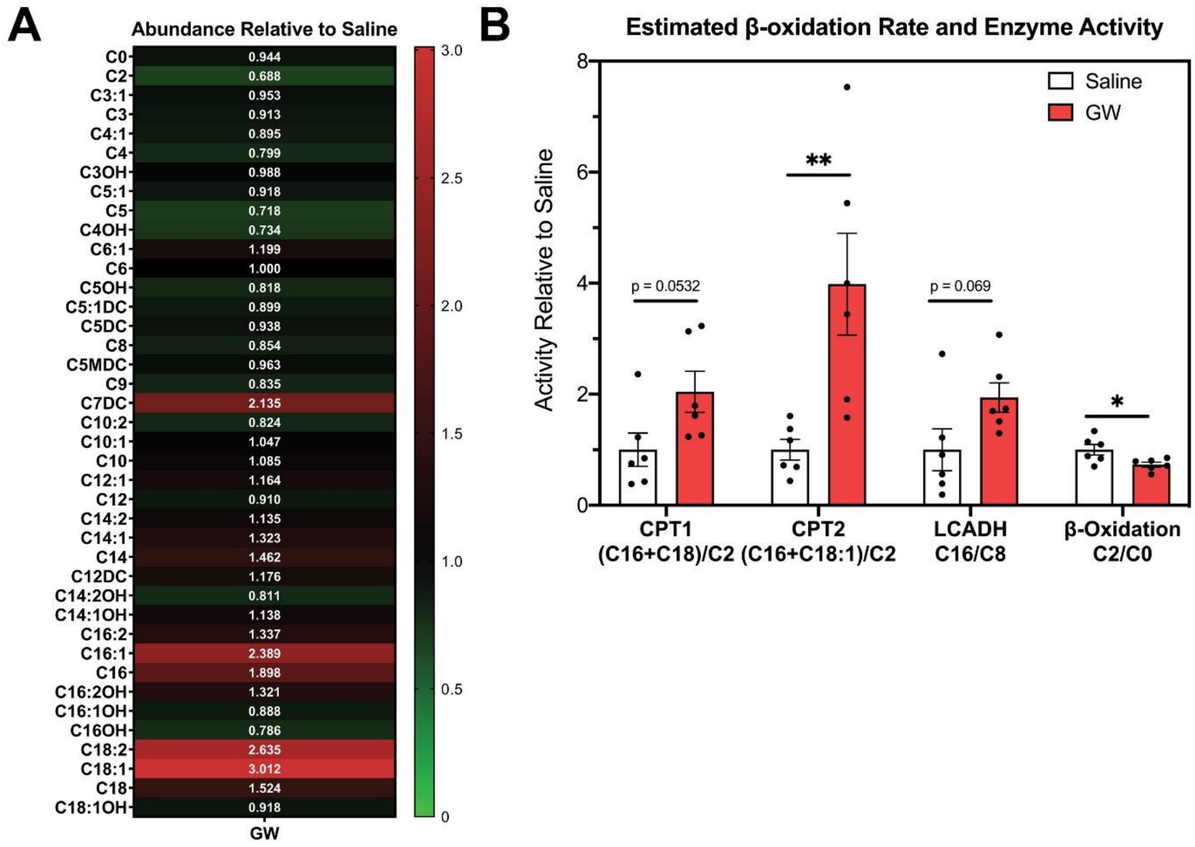

Appendix Figure S8: GW alters acylcarnitine metabolism and β-oxidation in mice.

Male BALB/C mice were injected subcutaneously with saline. After 5 days and everyday thereafter, the mice were injected with either saline or GW 5mg kg^{-1}. After 16 days, mice were euthanized and the metabolome of tibialis anterior muscles was analyzed through LC-MS/MS.

(A) Heatmap visualizing mean concentration of acylcarnitines relative to saline controls. Red and green indicate an increase or decrease in metabolite levels, respectively.

(B) Relative estimated activity of CPT1, CPT2, LCADH, and β-oxidation.

Data information: Individual data points represent values from individual mice, with a total of six mice per cohort (n = 6) Error bars represent the standard deviation (SD) of the mean. p-values were calculated with an ANOVA followed by Fisher's LSD test (*P < 0.05; **P < 0.01; ***P < 0.001).

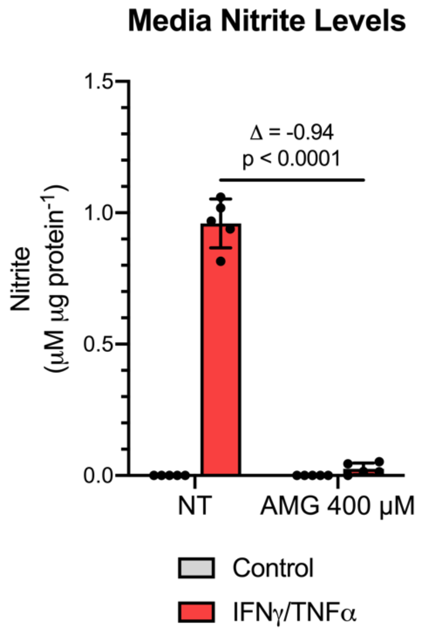

Appendix Figure S9: AMG prevents nitric oxide production in cytokine-treated myotubes.

C2C12 myotubes were treated with or without IFNγ (100U/mL) and TNFα (20ng/mL) and the indicated dose of aminoguanidine (AMG). Media nitrite levels were then measured 24h after treatment.

Data Information: Individual data points represent five independent experimental replicates (n=5). Error bars represent the standard deviation (SD) of the mean. For statistical comparisons, Δ indicates the difference of mean values and p-values were calculated with an ANOVA followed by Fisher's LSD test.

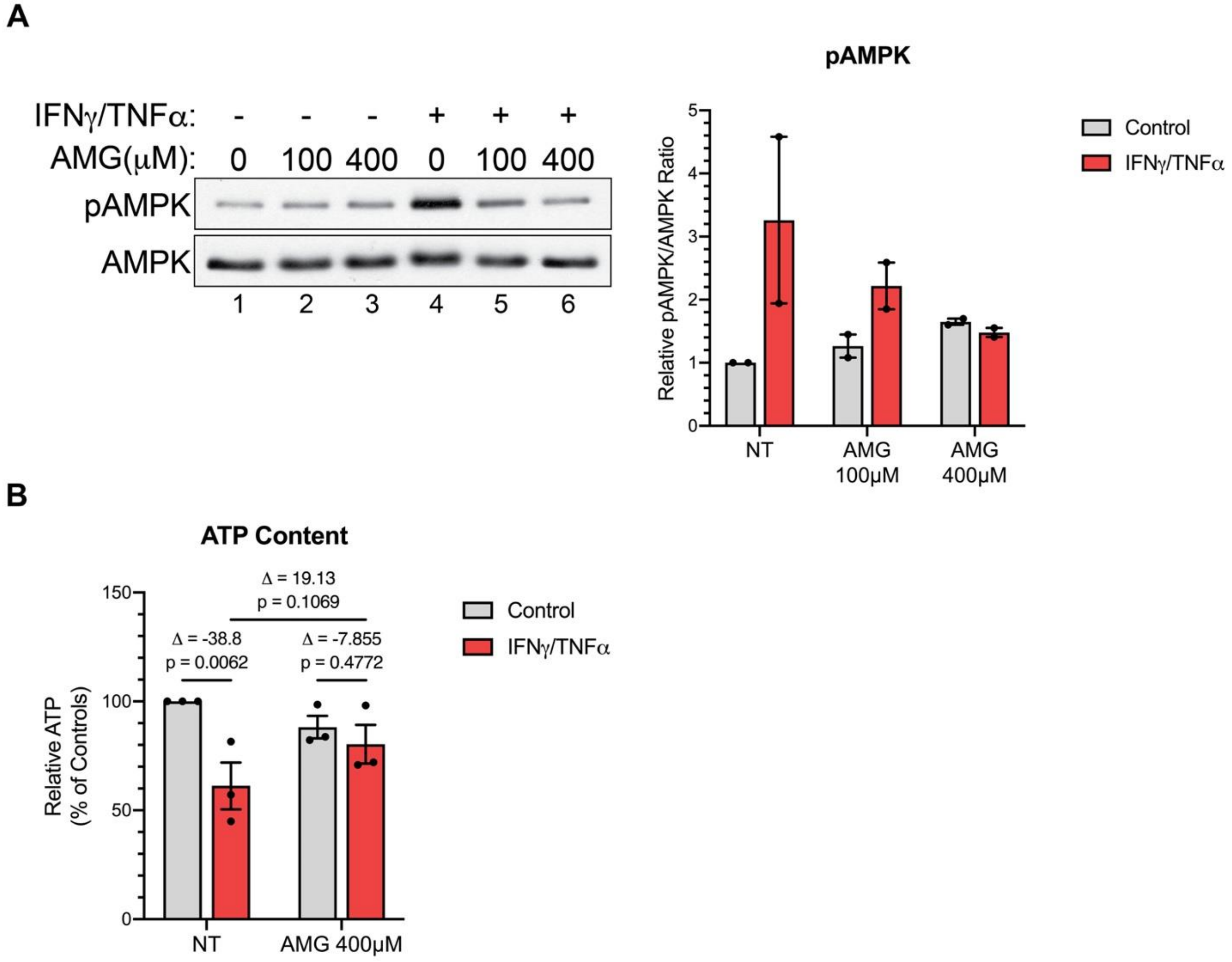

Appendix Figure S10: AMG recovers cellular ATP levels and reduces AMPK activation in cytokine-treated myotubes.

C2C12 myotubes were treated with or without IFNγ (100U/mL) and TNFα (20ng/mL) and the indicated doses aminoguanidine (AMG) for 24h.

(A) (*left*) Western blot analysis for pThr172-AMPK (pAMPK) and total AMPK (AMPK). (*right*) Quantification of the pAMPK to AMPK ratio relative to the untreated control (n=2).

(B) Cellular ATP content in myotubes treated with or without IFNγ and TNFα in the presence or absence of AMG. ATP content is shown as a percentage of the corresponding untreated control (n=3).

Data information: Individual data points are from two to three independent experimental replicates (n = 2-3). Error bars represent the standard deviation (SD) of the mean. For statistical comparisons, Δ indicates the difference of mean values and p-values were calculated with an ANOVA followed by Fisher's LSD test.

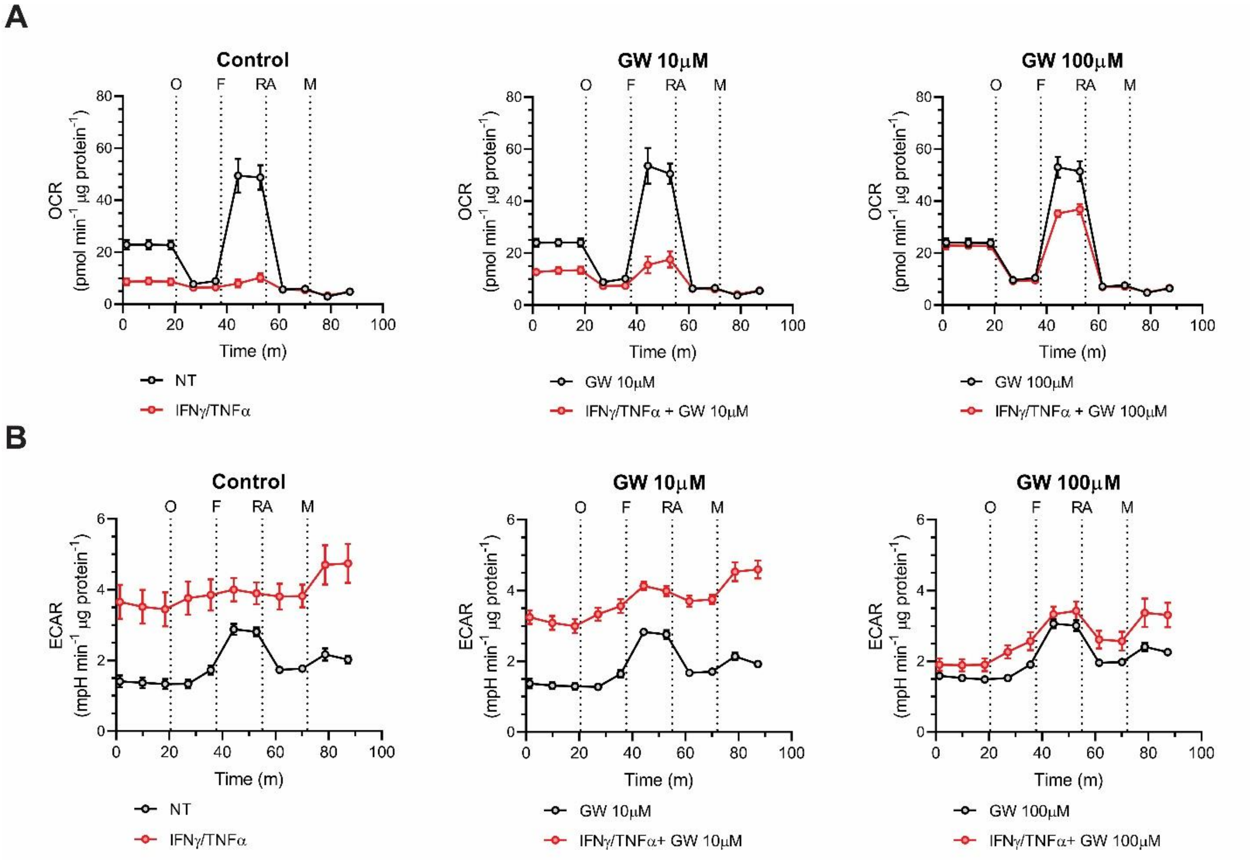

Appendix Figure S11: GW274150 reverses IFNγ/TNFα-induced suppression of OCR and elevation of ECAR.

C2C12 myotubes were treated with or without IFNγ (100U/mL) and TNFα (20ng/mL) and the indicated doses of GW274150 (GW). Extracellular flux was measured after 24h with a Seahorse XF Analyzer. Sequential injections of oligomycin (O, 1µM), FCCP (F, 1.5µM), Rotenone + Antimycin A (RA, 1µM), and monensin (M, 20µM) were performed to assess the bioenergetic profile.

(A) Oxygen consumption rate (OCR).

(B) Extracellular acidification rate (ECAR).

Data Information: Results are a representative example of three independent experiments (n = 3). Error bars represent the standard deviation of technical triplicates.

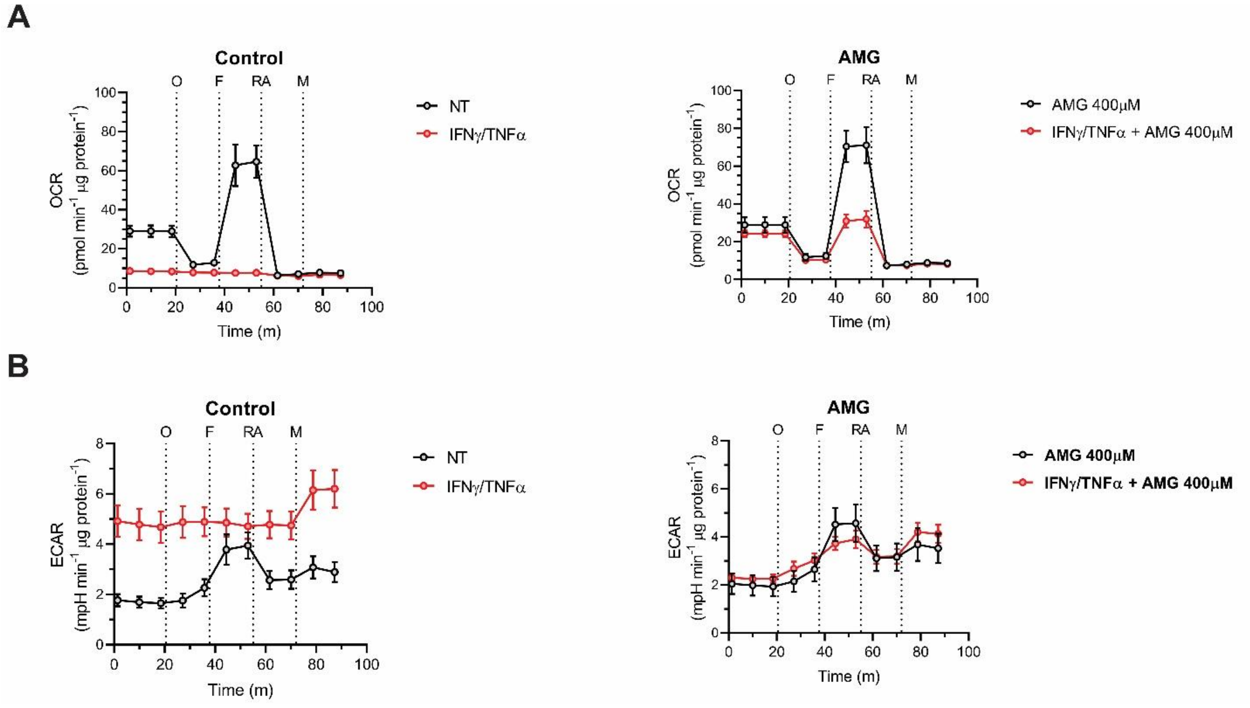

Appendix Figure S12: Aminoguanidine reverses IFNγ/TNFα-induced suppression of OCR and elevation of ECAR.

C2C12 myotubes were treated with or without IFNγ (100U/mL) and TNFα (20ng/mL) and aminoguanidine (AMG; 400µM). Extracellular flux was measured after 24h with a Seahorse XF analyzer. Sequential injections of oligomycin (O, 1µM), FCCP (F, 1.5µM), Rotenone + Antimycin A (RA, 1µM), and monensin (M, 20µM) were performed to assess the bioenergetic profile.

(A) Oxygen consumption rate (OCR).

(B) Extracellular acidification rate (ECAR).

Data Information: Results are a representative example of three independent experiments (n = 3). Error bars represent the standard deviation of technical replicates.

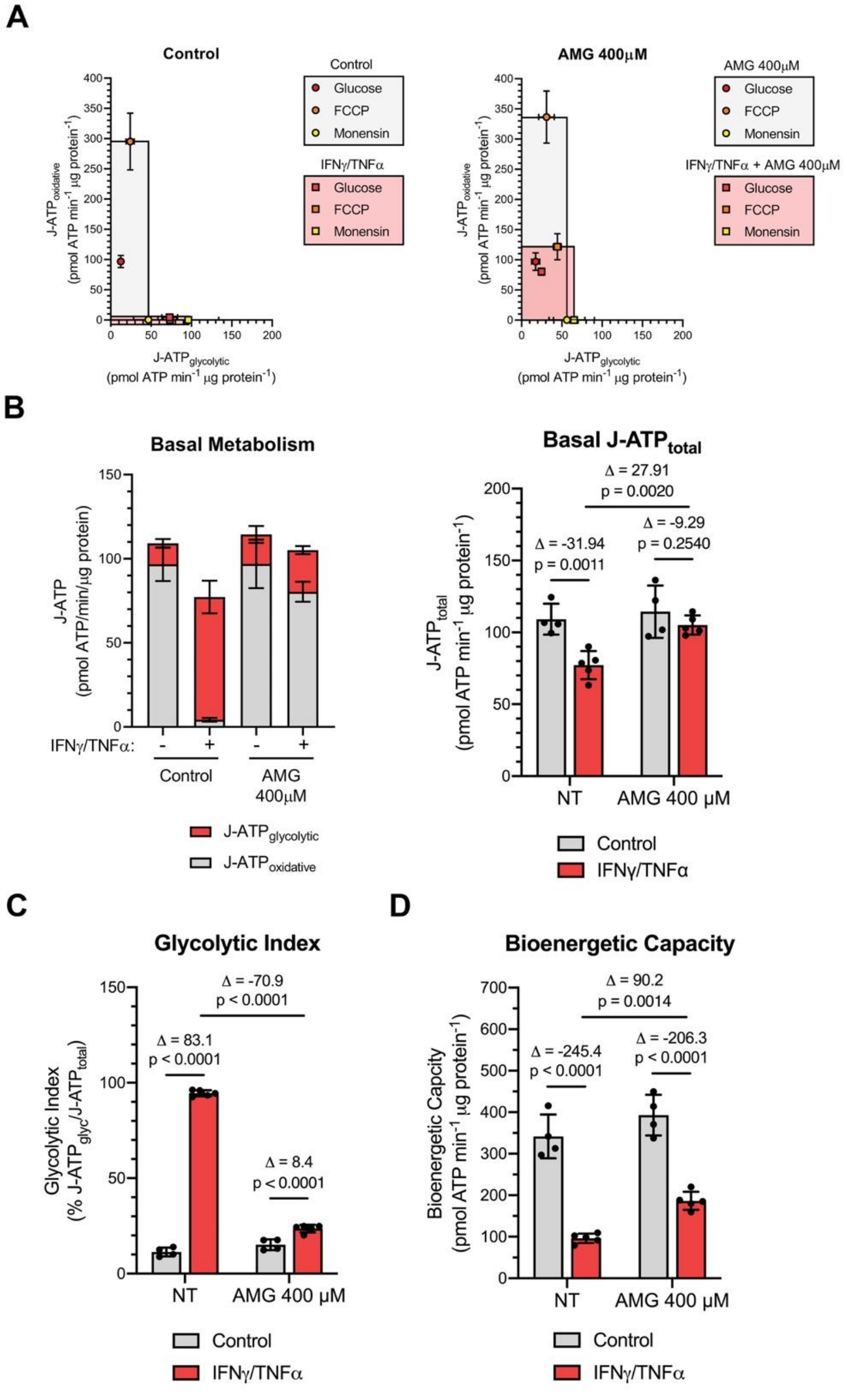

Appendix Figure S13: Aminoguanidine prevents a cytokine-induced shift to aerobic glycolysis in C2C12.

C2C12 myotubes were treated with or without IFNγ (100U/mL) and TNFα (20ng/mL) and aminoguanidine (AMG; 400μM). ATP production rates (J-ATP) from oxidative phosphorylation (oxidative) and glycolysis (glycolytic) were determined from measurements of extracellular flux 24h after treatment.

(A) Bioenergetic profiles. Highlighted squares are defined by the theoretical maximal J-ATP$_{oxidative}$ and J-ATP$_{glycolytic}$ rates.

(B) (*left)* Basal J-ATP$_{glycolytic}$ and J-ATP$_{oxidative}$ rates. (*right*) Total basal J-ATP rate.

(C) Glycolytic index of basal metabolism.

(D) Total bioenergetic capacity.

Data Information: Individual data points represent technical replicates. The data is representative of three independent experiments (n = 3). Error bars represent the standard deviation (SD) of the mean of technical replicates. For statistical comparisons, Δ indicates the difference of mean values of technical replicates and p-values were calculated with an ANOVA followed by Fisher's LSD test.

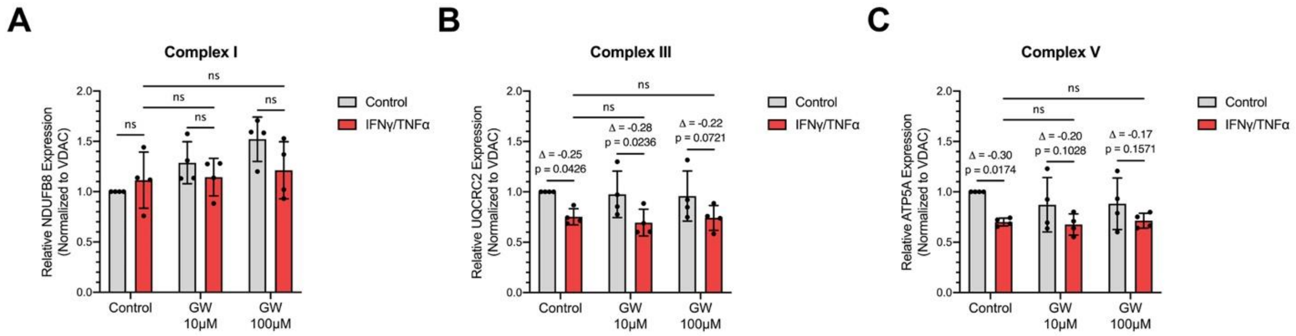

Appendix Figure S14: Effect of cytokine-induced muscle wasting on complex integrity.

C2C12 myotubes were treated with or without IFNγ (100U/mL) and TNFα (20ng/mL) and the indicated doses of GW274150 (GW). Protein content was extracted 24h after treatment.

(A-C) Quantification of complex subunits normalized to VDAC (Outer Mitochondrial Membrane; OMM) and relative to untreated control. (A) NDUFB8 (Complex I; CI) (B) UQCRC2 (Complex III; CIII) (C) ATP5A (Complex V; CV).

Data information: Individual data points are from four independent experimental replicates (n = 4). Error bars represent the standard deviation (SD) of the mean. For statistical comparisons, Δ indicates the difference of mean values and p-values were calculated with an ANOVA followed by Fisher's LSD test. Non statistically significant comparisons (P > 0.05) are indicated as non-significant (ns).

Appendix Tables

Appendix Table S1. Significance p-values for Figure 1.

Figure	Panel	Comparison	p-value	Significant?
1	B, right	WT Saline vs WT LPS	0.069	No
1	B, right	KO Saline vs KO LPS	0.6325	No
1	D	WT Saline vs. WT LPS	0.002	Yes
1	D	WT Saline vs. KO Saline	0.4889	No
1	D	WT Saline vs. KO LPS	0.3809	No
1	D	WT LPS vs. KO Saline	0.0135	Yes
1	D	WT LPS vs. KO LPS	0.0215	Yes
1	D	KO Saline vs. KO LPS	0.8527	No
1	E	WT Saline vs. WT LPS	0.0093	Yes
1	E	WT Saline vs. KO Saline	0.0637	No
1	E	WT Saline vs. KO LPS	0.3383	No
1	E	WT LPS vs. KO Saline	0.4261	No
1	E	WT LPS vs. KO LPS	0.0896	No
1	E	KO Saline vs. KO LPS	0.3585	No
1	F	WT Saline vs. WT LPS	0.0173	Yes
1	F	WT Saline vs. KO Saline	0.1215	No
1	F	WT Saline vs. KO LPS	0.0075	Yes
1	F	WT LPS vs. KO Saline	0.3825	No
1	F	WT LPS vs. KO LPS	0.7483	No
1	F	KO Saline vs. KO LPS	0.2342	No
1	G, right	WT Saline vs WT LPS	<0.0001	Yes
1	G, right	KO Saline vs KO LPS	0.3161	No
1	H	WT Saline vs. WT LPS	<0.0001	Yes
1	H	WT Saline vs. KO Saline	0.912	No
1	H	WT Saline vs. KO LPS	0.6808	No
1	H	WT LPS vs. KO Saline	0.0001	Yes
1	H	WT LPS vs. KO LPS	0.0003	Yes
1	H	KO Saline vs. KO LPS	0.6247	No

Abbreviations: WT, Wild-type. KO, iNOS Knockout. LPS, Lipopolysaccharide.

Appendix Table S2. Significance p-values for Figure 2.

Figure	Panel	Comparison	p-value	Significant?
2	B, Arginine biosynthesis	WT Saline vs WT LPS	0.000211	Yes
2	B, D-Glutamine and D-glutamate metabolism	WT Saline vs WT LPS	0.000717	Yes
2	B, Alanine, aspartate and glutamate metabolism	WT Saline vs WT LPS	0.001559	Yes
2	B, Citrate cycle (TCA cycle)	WT Saline vs WT LPS	0.001625	Yes
2	B, Aminoacyl-tRNA biosynthesis	WT Saline vs WT LPS	0.002228	Yes
2	B, Phenylalanine, tyrosine and tryptophan biosynthesis	WT Saline vs WT LPS	0.003177	Yes
2	B, Glycerophospholipid metabolism	WT Saline vs WT LPS	0.004314	Yes
2	B, Phenylalanine metabolism	WT Saline vs WT LPS	0.004961	Yes
2	B, Arginine and proline metabolism	WT Saline vs WT LPS	0.00933	Yes
2	B, Glyoxylate and dicarboxylate metabolism	WT Saline vs WT LPS	0.012332	Yes
2	B, Glycolysis / Gluconeogenesis	WT Saline vs WT LPS	0.030671	Yes
2	B, Pyruvate metabolism	WT Saline vs WT LPS	0.057666	No
2	B, Glycine, serine and threonine metabolism	WT Saline vs WT LPS	0.073631	No
2	B, Tyrosine metabolism	WT Saline vs WT LPS	0.083293	No
2	B, Starch and sucrose metabolism	WT Saline vs WT LPS	0.090833	No
2	B, Glutathione metabolism	WT Saline vs WT LPS	0.10425	No
2	B, Histidine metabolism	WT Saline vs WT LPS	0.12032	No
2	B, Tryptophan metabolism	WT Saline vs WT LPS	0.16151	No
2	B, Cysteine and methionine metabolism	WT Saline vs WT LPS	0.40348	No
2	B, Taurine and hypotaurine metabolism	WT Saline vs WT LPS	0.47049	No
2	B, beta-Alanine metabolism	WT Saline vs WT LPS	0.52032	No
2	B, Arginine biosynthesis	KO Saline vs KO LPS	0.067745	No
2	B, D-Glutamine and D-glutamate metabolism	KO Saline vs KO LPS	0.41731	No
2	B, Alanine, aspartate and glutamate metabolism	KO Saline vs KO LPS	0.29741	No
2	B, Citrate cycle (TCA cycle)	KO Saline vs KO LPS	0.41669	No
2	B, Aminoacyl-tRNA biosynthesis	KO Saline vs KO LPS	0.064105	No
2	B, Phenylalanine, tyrosine and tryptophan biosynthesis	KO Saline vs KO LPS	0.026998	Yes
2	B, Glycerophospholipid metabolism	KO Saline vs KO LPS	0.43848	No
2	B, Phenylalanine metabolism	KO Saline vs KO LPS	0.027012	Yes
2	B, Arginine and proline metabolism	KO Saline vs KO LPS	0.015403	Yes
2	B, Glyoxylate and dicarboxylate metabolism	KO Saline vs KO LPS	0.26029	No
2	B, Glycolysis / Gluconeogenesis	KO Saline vs KO LPS	0.45186	No
2	B, Pyruvate metabolism	KO Saline vs KO LPS	0.47224	No
2	B, Glycine, serine and threonine metabolism	KO Saline vs KO LPS	0.31681	No
2	B, Tyrosine metabolism	KO Saline vs KO LPS	0.09504	No
2	B, Starch and sucrose metabolism	KO Saline vs KO LPS	0.24164	No
2	B, Glutathione metabolism	KO Saline vs KO LPS	0.076092	No
2	B, Histidine metabolism	KO Saline vs KO LPS	0.4486	No
2	B, Tryptophan metabolism	KO Saline vs KO LPS	0.016627	Yes
2	B, Cysteine and methionine metabolism	KO Saline vs KO LPS	0.75813	No
2	B, Taurine and hypotaurine metabolism	KO Saline vs KO LPS	0.74198	No
2	B, beta-Alanine metabolism	KO Saline vs KO LPS	0.41639	No
2	C, right	WT Saline vs WT LPS	0.003	Yes
2	C, right	KO Saline vs KO LPS	<0.0001	Yes
2	E, Glucose	WT Saline vs WT LPS	0.0908	No
2	E, Glucose	KO Saline vs KO LPS	0.2416	No
2	E, Pyruvate	WT Saline vs WT LPS	0.095	No
2	E, Pyruvate	KO Saline vs KO LPS	0.5284	No
2	E, Citrate	WT Saline vs WT LPS	0.0226	Yes
2	E, Citrate	KO Saline vs KO LPS	0.2141	No
2	E, α-ketoglutarate	WT Saline vs WT LPS	0.0013	Yes
2	E, α-ketoglutarate	KO Saline vs KO LPS	0.8498	No
2	E, Succinate	WT Saline vs WT LPS	0.1263	No

2	E, Succinate	KO Saline vs KO LPS	0.1646	No
2	F, Arginine	WT Saline vs WT LPS	0.0151	Yes
2	F, Arginine	KO Saline vs KO LPS	0.6179	No
2	H, CPT1	WT Saline vs WT LPS	0.0135	Yes
2	H, CPT1	KO Saline vs KO LPS	0.2961	No
2	H, CPT2	WT Saline vs WT LPS	0.1369	No
2	H, CPT2	KO Saline vs KO LPS	0.923	No
2	H, LCADH	WT Saline vs WT LPS	0.5731	No
2	H, LCADH	KO Saline vs KO LPS	0.1448	No
2	H, β-Oxidation	WT Saline vs WT LPS	0.5692	No
2	H, β-Oxidation	KO Saline vs KO LPS	0.4015	No

Abbreviations: WT, Wild-type. KO, iNOS Knockout. LPS, Lipopolysaccharide.

Appendix Table S3. Significance p-values for Figure 3.

Figure	Panel	Comparison	p-value	Significant?
3	A	C26 vs C26 + GW	0.4498	No
3	B, right	Saline vs. C26	<0.0001	Yes
3	B, right	Saline vs. GW	0.0748	No
3	B, right	Saline vs. GW C26	0.0054	Yes
3	B, right	C26 vs. GW	<0.0001	Yes
3	B, right	C26 vs. GW C26	0.001	Yes
3	B, right	GW vs. GW C26	0.0001	Yes
3	C	Saline vs. C26	<0.0001	Yes
3	C	Saline vs. GW	0.6877	No
3	C	Saline vs. C26 + GW	<0.0001	Yes
3	C	C26 vs. GW	<0.0001	Yes
3	C	C26 vs. C26 + GW	0.0678	No
3	C	GW vs. C26 + GW	<0.0001	Yes
3	D	Saline vs. C26	<0.0001	Yes
3	D	Saline vs. GW	0.5405	No
3	D	Saline vs. C26 + GW	0.0001	Yes
3	D	C26 vs. GW	<0.0001	Yes
3	D	C26 vs. C26 + GW	0.0008	Yes
3	D	GW vs. C26 + GW	0.0006	Yes
3	E	Saline vs. C26	<0.0001	Yes
3	E	Saline vs. GW	0.0489	Yes
3	E	Saline vs. C26 + GW	<0.0001	Yes
3	E	C26 vs. GW	<0.0001	Yes
3	E	C26 vs. C26 + GW	0.0004	Yes
3	E	GW vs. C26 + GW	<0.0001	Yes
3	F	Saline vs. C26	<0.0001	Yes
3	F	Saline vs. GW	0.3933	No
3	F	Saline vs. C26 + GW	<0.0001	Yes
3	F	C26 vs. GW	<0.0001	Yes
3	F	C26 vs. C26 + GW	0.0051	Yes
3	F	GW vs. C26 + GW	<0.0001	Yes
3	G	Saline vs. C26	<0.0001	Yes
3	G	Saline vs. GW	0.5787	No
3	G	Saline vs. C26 + GW	0.0093	Yes
3	G	C26 vs. GW	0.0003	Yes
3	G	C26 vs. C26 + GW	0.0489	Yes
3	G	GW vs. C26 + GW	0.0314	Yes
3	H, right	Saline vs C26	<0.0001	Yes
3	H, right	Saline vs GW	0.1197	No
3	H, right	Saline vs C26 + GW	<0.0001	Yes
3	H, right	GW vs C26 + GW	<0.0001	Yes

3	H, right	C26 vs C26 + GW	<0.0001	Yes
3	I	Saline vs. C26	0.0009	Yes
3	I	Saline vs. GW	0.1805	No
3	I	Saline vs. C26 + GW	0.0306	Yes
3	I	C26 vs. GW	0.021	Yes
3	I	C26 vs. C26 + GW	0.1333	No
3	I	GW vs. C26 + GW	0.3588	No

Abbreviations: GW, GW274150.

Appendix Table S4. Significance p-values for Figure 4.

Figure	Panel	Comparison	p-value	Significant?
4	B, Arginine and proline metabolism	Saline vs C26	0.0000763	Yes
4	B, Cysteine and methionine metabolism	Saline vs C26	0.00017348	Yes
4	B, Histidine metabolism	Saline vs C26	0.0018642	Yes
4	B, Aminoacyl-tRNA biosynthesis	Saline vs C26	0.002167	Yes
4	B, Arginine biosynthesis	Saline vs C26	0.0029727	Yes
4	B, Pyruvate metabolism	Saline vs C26	0.0051888	Yes
4	B, Glycine, serine and threonine metabolism	Saline vs C26	0.006199	Yes
4	B, Glutathione metabolism	Saline vs C26	0.006411	Yes
4	B, Glycolysis / Gluconeogenesis	Saline vs C26	0.007385	Yes
4	B, Tryptophan metabolism	Saline vs C26	0.010722	Yes
4	B, beta-Alanine metabolism	Saline vs C26	0.025259	Yes
4	B, Glycerophospholipid metabolism	Saline vs C26	0.027221	Yes
4	B, Alanine, aspartate and glutamate metabolism	Saline vs C26	0.053877	No
4	B, Tyrosine metabolism	Saline vs C26	0.063717	No
4	B, Glyoxylate and dicarboxylate metabolism	Saline vs C26	0.088995	No
4	B, Citrate cycle (TCA cycle)	Saline vs C26	0.092513	No
4	B, D-Glutamine and D-glutamate metabolism	Saline vs C26	0.1418	No
4	B, Taurine and hypotaurine metabolism	Saline vs C26	0.25613	No
4	B, Phenylalanine, tyrosine and tryptophan biosynthesis	Saline vs C26	0.44076	No
4	B, Phenylalanine metabolism	Saline vs C26	0.51796	No
4	B, Starch and sucrose metabolism	Saline vs C26	0.86946	No
4	B, Arginine and proline metabolism	C26 vs C26 + GW	0.022394	Yes
4	B, Cysteine and methionine metabolism	C26 vs C26 + GW	0.07598	No
4	B, Histidine metabolism	C26 vs C26 + GW	0.17476	No
4	B, Aminoacyl-tRNA biosynthesis	C26 vs C26 + GW	0.052388	No
4	B, Arginine biosynthesis	C26 vs C26 + GW	0.031137	Yes
4	B, Pyruvate metabolism	C26 vs C26 + GW	0.013299	Yes
4	B, Glycine, serine and threonine metabolism	C26 vs C26 + GW	0.1443	No
4	B, Glutathione metabolism	C26 vs C26 + GW	0.048382	Yes
4	B, Glycolysis / Gluconeogenesis	C26 vs C26 + GW	0.032564	Yes
4	B, Tryptophan metabolism	C26 vs C26 + GW	0.080335	No
4	B, beta-Alanine metabolism	C26 vs C26 + GW	0.14921	No
4	B, Glycerophospholipid metabolism	C26 vs C26 + GW	0.122	No
4	B, Alanine, aspartate and glutamate metabolism	C26 vs C26 + GW	0.017309	Yes
4	B, Tyrosine metabolism	C26 vs C26 + GW	0.034933	Yes
4	B, Glyoxylate and dicarboxylate metabolism	C26 vs C26 + GW	0.11468	No
4	B, Citrate cycle (TCA cycle)	C26 vs C26 + GW	0.0051395	Yes
4	B, D-Glutamine and D-glutamate metabolism	C26 vs C26 + GW	0.29282	No
4	B, Taurine and hypotaurine metabolism	C26 vs C26 + GW	0.46719	No
4	B, Phenylalanine, tyrosine and tryptophan biosynthesis	C26 vs C26 + GW	0.099194	No
4	B, Phenylalanine metabolism	C26 vs C26 + GW	0.13622	No
4	B, Starch and sucrose metabolism	C26 vs C26 + GW	0.0000345	Yes
4	C, right	Saline vs. C26	0.0111	Yes
4	C, right	Saline vs. GW	0.8661	No
4	C, right	Saline vs. GW C26	0.228	No
4	C, right	C26 vs. GW	0.0086	Yes
4	C, right	C26 vs. GW C26	0.0831	No
4	C, right	GW vs. GW C26	0.1772	No
4	D, Glucose	Saline vs. C26	0.8658	No
4	D, Glucose	Saline vs. C26 + GW	<0.0001	Yes
4	D, Glucose	C26 vs. C26 + GW	<0.0001	Yes
4	D, Pyruvate	Saline vs. C26	0.0266	Yes
4	D, Pyruvate	Saline vs. C26 + GW	0.1733	No

4	D, Pyruvate	C26 vs. C26 + GW	0.32	No
4	E, Arginine	Saline vs. C26	0.0002	Yes
4	E, Arginine	Saline vs. C26 + GW	0.0432	Yes
4	E, Arginine	C26 vs. C26 + GW	0.0211	Yes
4	E, Lysine	Saline vs. C26	0.0019	Yes
4	E, Lysine	Saline vs. C26 + GW	0.1724	No
4	E, Lysine	C26 vs. C26 + GW	0.0341	Yes
4	E, Methylhistidine	Saline vs. C26	0.0002	Yes
4	E, Methylhistidine	Saline vs. C26 + GW	0.1732	No
4	E, Methylhistidine	C26 vs. C26 + GW	0.0033	Yes
4	E, Tryptophan	Saline vs. C26	0.0002	Yes
4	E, Tryptophan	Saline vs. C26 + GW	0.1198	No
4	E, Tryptophan	C26 vs. C26 + GW	0.0052	Yes
4	E, Aspartate	Saline vs. C26	0.0688	No
4	E, Aspartate	Saline vs. C26 + GW	0.8977	No
4	E, Aspartate	C26 vs. C26 + GW	0.0873	No
4	F, C3	Saline vs. C26	0.0051	Yes
4	F, C3	Saline vs. C26 + GW	0.265	No
4	F, C3	C26 vs. C26 + GW	0.0508	No
4	F, C5	Saline vs. C26	0.1307	No
4	F, C5	Saline vs. C26 + GW	0.7402	No
4	F, C5	C26 vs. C26 + GW	0.0718	No
4	G, CPT1	Saline vs. C26	0.8755	No
4	G, CPT1	Saline vs. C26 + GW	0.0024	Yes
4	G, CPT1	C26 vs. C26 + GW	0.0018	Yes
4	G, CPT2	Saline vs. C26	0.7068	No
4	G, CPT2	Saline vs. C26 + GW	<0.0001	Yes
4	G, CPT2	C26 vs. C26 + GW	0.0001	Yes
4	G, LCADH	Saline vs. C26	0.001	Yes
4	G, LCADH	Saline vs. C26 + GW	0.8868	No
4	G, LCADH	C26 vs. C26 + GW	0.0013	Yes
4	G, β-Oxidation	Saline vs. C26	0.0708	No
4	G, β-Oxidation	Saline vs. C26 + GW	0.3957	No
4	G, β-Oxidation	C26 vs. C26 + GW	0.0129	Yes

Abbreviations: GW, GW274150.

Appendix Table S5. Significance p-values for Figure 5.

Figure	Panel	Comparison	p-value	Significant?
5	B	NT vs. GW100	0.6031	No
5	B	NT vs. IT	0.0007	Yes
5	B	NT vs. IT+GW100	0.8074	No
5	B	GW100 vs. IT	0.0017	Yes
5	B	GW100 vs. IT+GW100	0.4487	No
5	B	IT vs. IT+GW100	0.0004	Yes
5	C, bottom	NT vs. GW10	>0.9999	No
5	C, bottom	NT vs. GW100	0.9712	No
5	C, bottom	NT vs. IT	<0.0001	Yes
5	C, bottom	NT vs. IT+GW10	<0.0001	Yes
5	C, bottom	NT vs. IT+GW100	0.0789	No
5	C, bottom	GW10 vs. GW100	0.9712	No
5	C, bottom	GW10 vs. IT	<0.0001	Yes
5	C, bottom	GW10 vs. IT+GW10	<0.0001	Yes
5	C, bottom	GW10 vs. IT+GW100	0.0789	No
5	C, bottom	GW100 vs. IT	<0.0001	Yes
5	C, bottom	GW100 vs. IT+GW10	<0.0001	Yes
5	C, bottom	GW100 vs. IT+GW100	0.074	No
5	C, bottom	IT vs. IT+GW10	0.1302	No
5	C, bottom	IT vs. IT+GW100	0.0001	Yes
5	C, bottom	IT+GW10 vs. IT+GW100	0.0019	Yes
5	F, left	NT vs. GW10	0.691	No
5	F, left	NT vs. GW100	0.564	No
5	F, left	NT vs. IT	0.0147	Yes
5	F, left	NT vs. IT+GW10	0.0619	No
5	F, left	NT vs. IT+GW100	0.6281	No
5	F, left	GW10 vs. GW100	0.8554	No
5	F, left	GW10 vs. IT	0.0312	Yes
5	F, left	GW10 vs. IT+GW10	0.1245	No
5	F, left	GW10 vs. IT+GW100	0.9299	No
5	F, left	GW100 vs. IT	0.0438	Yes
5	F, left	GW100 vs. IT+GW10	0.1685	No
5	F, left	GW100 vs. IT+GW100	0.9249	No
5	F, left	IT vs. IT+GW10	0.4463	No
5	F, left	IT vs. IT+GW100	0.0368	Yes
5	F, left	IT+GW10 vs. IT+GW100	0.1443	No
5	F, right	NT vs. GW10	0.581	No
5	F, right	NT vs. GW100	0.4961	No
5	F, right	NT vs. IT	0.005	Yes
5	F, right	NT vs. IT+GW10	0.0164	Yes
5	F, right	NT vs. IT+GW100	0.2289	No

5	F, right	GW10 vs. GW100	0.8952	No
5	F, right	GW10 vs. IT	0.0143	Yes
5	F, right	GW10 vs. IT+GW10	0.0463	Yes
5	F, right	GW10 vs. IT+GW100	0.497	No
5	F, right	GW100 vs. IT	0.0183	Yes
5	F, right	GW100 vs. IT+GW10	0.0589	No
5	F, right	GW100 vs. IT+GW100	0.5819	No
5	F, right	IT vs. IT+GW10	0.5335	No
5	F, right	IT vs. IT+GW100	0.0515	No
5	F, right	IT+GW10 vs. IT+GW100	0.1542	No
5	G	NT vs. GW10	0.747	No
5	G	NT vs. GW100	0.2054	No
5	G	NT vs. IT	0.0174	Yes
5	G	NT vs. IT+GW10	0.125	No
5	G	NT vs. IT+GW100	0.5683	No
5	G	GW10 vs. GW100	0.333	No
5	G	GW10 vs. IT	0.0094	Yes
5	G	GW10 vs. IT+GW10	0.0712	No
5	G	GW10 vs. IT+GW100	0.802	No
5	G	GW100 vs. IT	0.0015	Yes
5	G	GW100 vs. IT+GW10	0.0113	Yes
5	G	GW100 vs. IT+GW100	0.4663	No
5	G	IT vs. IT+GW10	0.2901	No
5	G	IT vs. IT+GW100	0.0059	Yes
5	G	IT+GW10 vs. IT+GW100	0.0451	Yes
5	I, left	NT vs. GW10	0.3758	No
5	I, left	NT vs. GW100	0.3573	No
5	I, left	NT vs. IT	0.0001	Yes
5	I, left	NT vs. IT+GW10	0.0145	Yes
5	I, left	NT vs. IT+GW100	0.2896	No
5	I, left	GW10 vs. GW100	0.9715	No
5	I, left	GW10 vs. IT	0.0014	Yes
5	I, left	GW10 vs. IT+GW10	0.096	No
5	I, left	GW10 vs. IT+GW100	0.8583	No
5	I, left	GW100 vs. IT	0.0015	Yes
5	I, left	GW100 vs. IT+GW10	0.1027	No
5	I, left	GW100 vs. IT+GW100	0.8864	No
5	I, left	IT vs. IT+GW10	0.071	No
5	I, left	IT vs. IT+GW100	0.0021	Yes
5	I, left	IT+GW10 vs. IT+GW100	0.1337	No
5	I, right	NT vs. GW10	0.6155	No
5	I, right	NT vs. GW100	0.8606	No
5	I, right	NT vs. IT	0.0003	Yes

5	I, right	NT vs. IT+GW10	0.0098	Yes
5	I, right	NT vs. IT+GW100	0.1082	No
5	I, right	GW10 vs. GW100	0.4991	No
5	I, right	GW10 vs. IT	0.0012	Yes
5	I, right	GW10 vs. IT+GW10	0.0307	Yes
5	I, right	GW10 vs. IT+GW100	0.2576	No
5	I, right	GW100 vs. IT	0.0002	Yes
5	I, right	GW100 vs. IT+GW10	0.0065	Yes
5	I, right	GW100 vs. IT+GW100	0.0773	No
5	I, right	IT vs. IT+GW10	0.179	No
5	I, right	IT vs. IT+GW100	0.0188	Yes
5	I, right	IT+GW10 vs. IT+GW100	0.2672	No

Abbreviations: NT, non-treated. GW, GW274150. IT, IFNγ/TNFα.

Appendix Table S6. Significance p-values for Figure 6.

Figure	Panel	Comparison	p-value	Significant?
6	B, right	NT vs. GW10	0.392	No
6	B, right	NT vs. GW100	0.3495	No
6	B, right	NT vs. IT	<0.0001	Yes
6	B, right	NT vs. IT+GW10	0.016	Yes
6	B, right	NT vs. IT+GW100	0.2457	No
6	B, right	GW10 vs. GW100	0.0873	No
6	B, right	GW10 vs. IT	0.0001	Yes
6	B, right	GW10 vs. IT+GW10	0.0797	No
6	B, right	GW10 vs. IT+GW100	0.0567	No
6	B, right	GW100 vs. IT	<0.0001	Yes
6	B, right	GW100 vs. IT+GW10	0.0026	Yes
6	B, right	GW100 vs. IT+GW100	0.809	No
6	B, right	IT vs. IT+GW10	0.0038	Yes
6	B, right	IT vs. IT+GW100	<0.0001	Yes
6	B, right	IT+GW10 vs. IT+GW100	0.0017	Yes
6	C	NT vs. GW10	>0.9999	No
6	C	NT vs. GW100	0.4618	No
6	C	NT vs. IT	<0.0001	Yes
6	C	NT vs. IT+GW10	<0.0001	Yes
6	C	NT vs. IT+GW100	0.0321	Yes
6	C	GW10 vs. GW100	0.4618	No
6	C	GW10 vs. IT	<0.0001	Yes
6	C	GW10 vs. IT+GW10	<0.0001	Yes
6	C	GW10 vs. IT+GW100	0.0321	Yes
6	C	GW100 vs. IT	<0.0001	Yes
6	C	GW100 vs. IT+GW10	<0.0001	Yes
6	C	GW100 vs. IT+GW100	0.1222	No
6	C	IT vs. IT+GW10	0.001	Yes
6	C	IT vs. IT+GW100	<0.0001	Yes
6	C	IT+GW10 vs. IT+GW100	0.0003	Yes
6	D	NT vs. GW10	0.5644	No
6	D	NT vs. GW100	0.9346	No
6	D	NT vs. IT	<0.0001	Yes
6	D	NT vs. IT+GW10	0.001	Yes
6	D	NT vs. IT+GW100	0.0139	Yes
6	D	GW10 vs. GW100	0.5116	No
6	D	GW10 vs. IT	<0.0001	Yes
6	D	GW10 vs. IT+GW10	0.0028	Yes
6	D	GW10 vs. IT+GW100	0.0412	Yes
6	D	GW100 vs. IT	<0.0001	Yes
6	D	GW100 vs. IT+GW10	0.0008	Yes

6	D	GW100 vs. IT+GW100	0.0119	Yes
6	D	IT vs. IT+GW10	0.0478	Yes
6	D	IT vs. IT+GW100	0.0033	Yes
6	D	IT+GW10 vs. IT+GW100	0.1719	No

Abbreviations: NT, non-treated. GW, GW274150. IT, IFNγ/TNFα.

Appendix Table S7. Significance p-values for Figure 7.

Figure	Panel	Comparison	p-value	Significant?
7	B, left	NT vs. GW10	0.5404	No
7	B, left	NT vs. GW100	0.7032	No
7	B, left	NT vs. IT	<0.0001	Yes
7	B, left	NT vs. IT+GW10	0.0002	Yes
7	B, left	NT vs. IT+GW100	0.0398	Yes
7	B, left	GW10 vs. GW100	0.3253	No
7	B, left	GW10 vs. IT	0.0003	Yes
7	B, left	GW10 vs. IT+GW10	0.0008	Yes
7	B, left	GW10 vs. IT+GW100	0.1286	No
7	B, left	GW100 vs. IT	<0.0001	Yes
7	B, left	GW100 vs. IT+GW10	<0.0001	Yes
7	B, left	GW100 vs. IT+GW100	0.0179	Yes
7	B, left	IT vs. IT+GW10	0.612	No
7	B, left	IT vs. IT+GW100	0.0088	Yes
7	B, left	IT+GW10 vs. IT+GW100	0.0262	Yes
7	B, right	NT vs. GW10	0.4624	No
7	B, right	NT vs. GW100	0.112	No
7	B, right	NT vs. IT	<0.0001	Yes
7	B, right	NT vs. IT+GW10	<0.0001	Yes
7	B, right	NT vs. IT+GW100	0.0002	Yes
7	B, right	GW10 vs. GW100	0.3697	No
7	B, right	GW10 vs. IT	<0.0001	Yes
7	B, right	GW10 vs. IT+GW10	<0.0001	Yes
7	B, right	GW10 vs. IT+GW100	0.0011	Yes
7	B, right	GW100 vs. IT	<0.0001	Yes
7	B, right	GW100 vs. IT+GW10	<0.0001	Yes
7	B, right	GW100 vs. IT+GW100	0.0085	Yes
7	B, right	IT vs. IT+GW10	0.3868	No
7	B, right	IT vs. IT+GW100	<0.0001	Yes
7	B, right	IT+GW10 vs. IT+GW100	0.0003	Yes

Abbreviations: NT, non-treated. GW, GW274150. IT, IFNγ/TNFα.

Appendix Table S8. Significance p-values for Figure EV1.

Figure	Panel	Comparison	p-value	Significant?
EV1	A	WT Saline vs. WT LPS	<0.0001	Yes
EV1	A	WT Saline vs. KO Saline	0.7346	No
EV1	A	WT Saline vs. KO LPS	<0.0001	Yes
EV1	A	WT LPS vs. KO Saline	<0.0001	Yes
EV1	A	WT LPS vs. KO LPS	0.6921	No
EV1	A	KO Saline vs. KO LPS	<0.0001	Yes
EV1	C	WT Saline vs. WT LPS	0.0004	Yes
EV1	C	WT Saline vs. KO Saline	0.7716	No
EV1	C	WT Saline vs. KO LPS	0.0013	Yes
EV1	C	WT LPS vs. KO Saline	0.0018	Yes
EV1	C	WT LPS vs. KO LPS	0.904	No
EV1	C	KO Saline vs. KO LPS	0.0046	Yes
EV1	D	WT Saline vs. WT LPS	<0.0001	Yes
EV1	D	WT Saline vs. KO Saline	0.7536	No
EV1	D	WT Saline vs. KO LPS	0.0002	Yes
EV1	D	WT LPS vs. KO Saline	0.0002	Yes
EV1	D	WT LPS vs. KO LPS	0.7481	No
EV1	D	KO Saline vs. KO LPS	0.0008	Yes
EV1	E	WT Saline vs. WT LPS	0.0369	Yes
EV1	E	KO Saline vs. KO LPS	0.0601	No
EV1	F	WT Saline vs. WT LPS	0.3227	No
EV1	F	KO Saline vs. KO LPS	0.6345	No
EV1	G	WT Saline vs. WT LPS	0.472	No
EV1	G	KO Saline vs. KO LPS	0.5961	No
EV1	H	WT Saline vs. WT LPS	0.0279	Yes
EV1	H	KO Saline vs. KO LPS	0.0965	No
EV1	I	WT Saline vs. WT LPS	0.0008	Yes
EV1	I	KO Saline vs. KO LPS	0.0081	Yes

Abbreviations: WT, Wild-type. KO, iNOS Knockout. LPS, Lipopolysaccharide.

Appendix Table S9. Significance p-values for Figure EV2.

Figure	Panel	Comparison	p-value	Significant?
EV2	A	WT Saline vs. WT LPS	0.2982	No
EV2	A	WT Saline vs. KO Saline	0.9812	No
EV2	A	WT Saline vs. KO LPS	0.0618	No
EV2	A	WT LPS vs. KO Saline	0.3091	No
EV2	A	WT LPS vs. KO LPS	0.3956	No
EV2	A	KO Saline vs. KO LPS	0.065	No
EV2	B	WT Saline vs. WT LPS	0.9479	No
EV2	B	WT Saline vs. KO Saline	0.7501	No
EV2	B	WT Saline vs. KO LPS	0.4338	No
EV2	B	WT LPS vs. KO Saline	0.8001	No
EV2	B	WT LPS vs. KO LPS	0.4729	No
EV2	B	KO Saline vs. KO LPS	0.6414	No
EV2	C	WT Saline vs. WT LPS	0.0017	Yes
EV2	C	KO Saline vs. KO LPS	0.4854	No

Abbreviations: WT, Wild-type. KO, iNOS Knockout. LPS, Lipopolysaccharide.

Appendix Table S10. Significance p-values for Figure EV3.

Figure	Panel	Comparison	p-value	Significant?
EV3	B	Saline vs. C26	<0.0001	Yes
EV3	B	Saline vs. GW	0.9261	No
EV3	B	Saline vs. C26 + GW	<0.0001	Yes
EV3	B	C26 vs. GW	<0.0001	Yes
EV3	B	C26 vs. C26 + GW	0.9596	No
EV3	B	GW vs. C26 + GW	<0.0001	Yes
EV3	C	Saline vs. C26	0.0133	Yes
EV3	C	Saline vs. GW	0.3978	No
EV3	C	Saline vs. C26 + GW	0.0007	Yes
EV3	C	C26 vs. GW	0.0736	No
EV3	C	C26 vs. C26 + GW	0.1782	No
EV3	C	GW vs. C26 + GW	0.0043	Yes
EV3	D	Saline vs. C26	0.3978	No
EV3	D	Saline vs. GW	0.4581	No
EV3	D	Saline vs. C26 + GW	0.4926	No
EV3	D	C26 vs. GW	0.1208	No
EV3	D	C26 vs. C26 + GW	0.8706	No
EV3	D	GW vs. C26 + GW	0.1611	No
EV3	E	Saline vs. C26	0.0002	Yes
EV3	E	Saline vs. GW	0.4356	No
EV3	E	Saline vs. C26 + GW	0.6234	No
EV3	E	C26 vs. GW	<0.0001	Yes
EV3	E	C26 vs. C26 + GW	0.0006	Yes
EV3	E	GW vs. C26 + GW	0.2103	No
EV3	F	Saline vs. C26	0.0002	Yes
EV3	F	Saline vs. GW	0.4821	No
EV3	F	Saline vs. C26 + GW	0.184	No
EV3	F	C26 vs. GW	0.0013	Yes
EV3	F	C26 vs. C26 + GW	0.006	Yes
EV3	F	GW vs. C26 + GW	0.5168	No
EV3	G	Saline vs. C26	0.1511	No
EV3	G	Saline vs. GW	0.3265	No
EV3	G	Saline vs. C26 + GW	0.7186	No
EV3	G	C26 vs. GW	0.0219	Yes
EV3	G	C26 vs. C26 + GW	0.105	No
EV3	G	GW vs. C26 + GW	0.5985	No
EV3	H	Saline vs. C26	0.0004	Yes
EV3	H	Saline vs. GW	0.1141	No
EV3	H	Saline vs. C26 + GW	0.0002	Yes
EV3	H	C26 vs. GW	0.0173	Yes
EV3	H	C26 vs. C26 + GW	0.8213	No

EV3	H	GW vs. C26 + GW	0.0105	Yes
EV3	I	Saline vs C26	<0.0001	Yes
EV3	I	Saline vs GW	0.0022	Yes
EV3	I	Saline vs C26 + GW	<0.0001	Yes
EV3	I	GW vs C26 + GW	<0.0001	Yes
EV3	I	C26 vs C26 + GW	<0.0001	Yes

Abbreviations: GW, GW274150.

Appendix Table S11. Significance p-values for Figure EV4.

Figure	Panel	Comparison	p-value	Significant?
EV4	B	NT vs. AMG100	0.2743	No
EV4	B	NT vs. AMG400	0.7906	No
EV4	B	NT vs. IT	0.1465	No
EV4	B	NT vs. IT+AMG100	0.7981	No
EV4	B	NT vs. IT+AMG400	0.9045	No
EV4	B	AMG100 vs. AMG400	0.3993	No
EV4	B	AMG100 vs. IT	0.0194	Yes
EV4	B	AMG100 vs. IT+AMG100	0.3941	No
EV4	B	AMG100 vs. IT+AMG400	0.3265	No
EV4	B	AMG400 vs. IT	0.0931	No
EV4	B	AMG400 vs. IT+AMG100	0.9922	No
EV4	B	AMG400 vs. IT+AMG400	0.884	No
EV4	B	IT vs. IT+AMG100	0.0947	No
EV4	B	IT vs. IT+AMG400	0.1197	No
EV4	B	IT+AMG100 vs. IT+AMG400	0.8917	No
EV4	C	NT vs. AMG100	0.7115	No
EV4	C	NT vs. AMG400	0.1703	No
EV4	C	NT vs. IT	<0.0001	Yes
EV4	C	NT vs. IT+AMG100	0.0009	Yes
EV4	C	NT vs. IT+AMG400	0.1432	No
EV4	C	AMG100 vs. AMG400	0.091	No
EV4	C	AMG100 vs. IT	<0.0001	Yes
EV4	C	AMG100 vs. IT+AMG100	0.0005	Yes
EV4	C	AMG100 vs. IT+AMG400	0.0755	No
EV4	C	AMG400 vs. IT	0.0002	Yes
EV4	C	AMG400 vs. IT+AMG100	0.0126	Yes
EV4	C	AMG400 vs. IT+AMG400	0.9161	No
EV4	C	IT vs. IT+AMG100	0.0474	Yes
EV4	C	IT vs. IT+AMG400	0.0003	Yes
EV4	C	IT+AMG100 vs. IT+AMG400	0.0154	Yes
EV4	D	NT vs. AMG100	0.5085	No
EV4	D	NT vs. AMG400	0.8605	No
EV4	D	NT vs. IT	0.6102	No
EV4	D	NT vs. IT+AMG100	0.5198	No
EV4	D	NT vs. IT+AMG400	0.1709	No
EV4	D	AMG100 vs. AMG400	0.6248	No
EV4	D	AMG100 vs. IT	0.2514	No
EV4	D	AMG100 vs. IT+AMG100	0.9856	No
EV4	D	AMG100 vs. IT+AMG400	0.4532	No
EV4	D	AMG400 vs. IT	0.4954	No
EV4	D	AMG400 vs. IT+AMG100	0.6374	No

EV4	D	AMG400 vs. IT+AMG400	0.2257	No
EV4	D	IT vs. IT+AMG100	0.2584	No
EV4	D	IT vs. IT+AMG400	0.0711	No
EV4	D	IT+AMG100 vs. IT+AMG400	0.4428	No
EV4	E	NT vs. AMG100	0.5051	No
EV4	E	NT vs. AMG400	0.0424	Yes
EV4	E	NT vs. IT	<0.0001	Yes
EV4	E	NT vs. IT+AMG100	0.0002	Yes
EV4	E	NT vs. IT+AMG400	0.2482	No
EV4	E	AMG100 vs. AMG400	0.1393	No
EV4	E	AMG100 vs. IT	<0.0001	Yes
EV4	E	AMG100 vs. IT+AMG100	0.0006	Yes
EV4	E	AMG100 vs. IT+AMG400	0.608	No
EV4	E	AMG400 vs. IT	<0.0001	Yes
EV4	E	AMG400 vs. IT+AMG100	0.0103	Yes
EV4	E	AMG400 vs. IT+AMG400	0.3114	No
EV4	E	IT vs. IT+AMG100	0.0033	Yes
EV4	E	IT vs. IT+AMG400	<0.0001	Yes
EV4	E	IT+AMG100 vs. IT+AMG400	0.0015	Yes
EV4	F	NT vs. AMG100	0.2547	No
EV4	F	NT vs. AMG400	0.4646	No
EV4	F	NT vs. IT	0.756	No
EV4	F	NT vs. IT+AMG100	0.3208	No
EV4	F	NT vs. IT+AMG400	0.4538	No
EV4	F	AMG100 vs. AMG400	0.6671	No
EV4	F	AMG100 vs. IT	0.3969	No
EV4	F	AMG100 vs. IT+AMG100	0.875	No
EV4	F	AMG100 vs. IT+AMG400	0.6804	No
EV4	F	AMG400 vs. IT	0.6695	No
EV4	F	AMG400 vs. IT+AMG100	0.784	No
EV4	F	AMG400 vs. IT+AMG400	0.9853	No
EV4	F	IT vs. IT+AMG100	0.4866	No
EV4	F	IT vs. IT+AMG400	0.6563	No
EV4	F	IT+AMG100 vs. IT+AMG400	0.7981	No

Abbreviations: NT, non-treated. AMG, Aminoguanidine. IT, IFNγ/TNFα.

Appendix Table S12. Significance p-values for Appendix Figure 4.

Figure	Panel	Comparison	p-value	Significant?
Appendix Fig 4	A	WT Saline vs. WT LPS	<0.0001	Yes
Appendix Fig 4	A	WT Saline vs. KO Saline	0.7367	No
Appendix Fig 4	A	WT Saline vs. KO LPS	<0.0001	Yes
Appendix Fig 4	A	WT Saline vs. WT LPS GW	<0.0001	Yes
Appendix Fig 4	A	WT LPS vs. KO Saline	<0.0001	Yes
Appendix Fig 4	A	WT LPS vs. KO LPS	0.6944	No
Appendix Fig 4	A	WT LPS vs. WT LPS GW	0.7026	No
Appendix Fig 4	A	KO Saline vs. KO LPS	<0.0001	Yes
Appendix Fig 4	A	KO Saline vs. WT LPS GW	<0.0001	Yes
Appendix Fig 4	A	KO LPS vs. WT LPS GW	0.9568	No
Appendix Fig 4	B	WT Saline vs. WT LPS	0.2828	No
Appendix Fig 4	B	WT Saline vs. KO Saline	0.9806	No
Appendix Fig 4	B	WT Saline vs. KO LPS	0.0537	No
Appendix Fig 4	B	WT Saline vs. WT LPS GW	0.0025	Yes
Appendix Fig 4	B	WT LPS vs. KO Saline	0.2937	No
Appendix Fig 4	B	WT LPS vs. KO LPS	0.3805	No
Appendix Fig 4	B	WT LPS vs. WT LPS GW	0.0284	Yes
Appendix Fig 4	B	KO Saline vs. KO LPS	0.0566	No
Appendix Fig 4	B	KO Saline vs. WT LPS GW	0.0027	Yes
Appendix Fig 4	B	KO LPS vs. WT LPS GW	0.1367	No
Appendix Fig 4	C	WT Saline vs. WT LPS	0.0014	Yes
Appendix Fig 4	C	WT Saline vs. KO Saline	0.477	No
Appendix Fig 4	C	WT Saline vs. KO LPS	0.3679	No
Appendix Fig 4	C	WT Saline vs. WT LPS GW	0.7929	No
Appendix Fig 4	C	WT LPS vs. KO Saline	0.011	Yes
Appendix Fig 4	C	WT LPS vs. KO LPS	0.018	Yes
Appendix Fig 4	C	WT LPS vs. WT LPS GW	0.0143	Yes
Appendix Fig 4	C	KO Saline vs. KO LPS	0.8487	No
Appendix Fig 4	C	KO Saline vs. WT LPS GW	0.7402	No
Appendix Fig 4	C	KO LPS vs. WT LPS GW	0.6239	No
Appendix Fig 4	D	WT Saline vs. WT LPS	<0.0001	Yes
Appendix Fig 4	D	WT Saline vs. KO Saline	0.906	No
Appendix Fig 4	D	WT Saline vs. KO LPS	0.6605	No
Appendix Fig 4	D	WT Saline vs. WT LPS GW	0.8179	No
Appendix Fig 4	D	WT LPS vs. KO Saline	<0.0001	Yes
Appendix Fig 4	D	WT LPS vs. KO LPS	0.0001	Yes
Appendix Fig 4	D	WT LPS vs. WT LPS GW	0.0002	Yes
Appendix Fig 4	D	KO Saline vs. KO LPS	0.6015	No
Appendix Fig 4	D	KO Saline vs. WT LPS GW	0.7465	No
Appendix Fig 4	D	KO LPS vs. WT LPS GW	0.8608	No

Abbreviations: WT, Wild-type. KO, iNOS Knockout. LPS, Lipopolysaccharide. GW, GW 274150

Appendix Table S13. Significance p-values for Appendix Figure 5.

Figure	Panel	Comparison	p-value	Significant?
Appendix Fig 5	A, Arginine biosynthesis	Saline vs GW	0.03538	Yes
Appendix Fig 5	A, D-Glutamine and D-glutamate metabolism	Saline vs GW	0.04436	Yes
Appendix Fig 5	A, Histidine metabolism	Saline vs GW	0.078932	No
Appendix Fig 5	A, Tyrosine metabolism	Saline vs GW	0.083479	No
Appendix Fig 5	A, Arginine and proline metabolism	Saline vs GW	0.093298	No
Appendix Fig 5	A, Cysteine and methionine metabolism	Saline vs GW	0.12038	No
Appendix Fig 5	A, Alanine, aspartate and glutamate metabolism	Saline vs GW	0.16577	No
Appendix Fig 5	A, Aminoacyl-tRNA biosynthesis	Saline vs GW	0.16687	No
Appendix Fig 5	A, Phenylalanine metabolism	Saline vs GW	0.17065	No
Appendix Fig 5	A, Citrate cycle (TCA cycle)	Saline vs GW	0.17229	No
Appendix Fig 5	A, Glutathione metabolism	Saline vs GW	0.17556	No
Appendix Fig 5	A, Glyoxylate and dicarboxylate metabolism	Saline vs GW	0.17565	No
Appendix Fig 5	A, Phenylalanine, tyrosine and tryptophan biosynthesis	Saline vs GW	0.2085	No
Appendix Fig 5	A, Glycine, serine and threonine metabolism	Saline vs GW	0.22846	No
Appendix Fig 5	A, Pyruvate metabolism	Saline vs GW	0.31862	No
Appendix Fig 5	A, Tryptophan metabolism	Saline vs GW	0.34651	No
Appendix Fig 5	A, Glycerophospholipid metabolism	Saline vs GW	0.39109	No
Appendix Fig 5	A, Taurine and hypotaurine metabolism	Saline vs GW	0.45686	No
Appendix Fig 5	A, beta-Alanine metabolism	Saline vs GW	0.48959	No
Appendix Fig 5	A, Glycolysis / Gluconeogenesis	Saline vs GW	0.52245	No

Abbreviations: GW, GW274150.

Appendix Table S14. Significance p-values for Appendix Figure 7.

Figure	Panel	Comparison	p-value	Significant?
Appendix Fig 7	B, C0	Saline vs. C26	0.0141	Yes
Appendix Fig 7	B, C0	Saline vs. C26 + GW	0.2325	No
Appendix Fig 7	B, C0	C26 vs. C26 + GW	0.1462	No
Appendix Fig 7	B, C10	Saline vs. C26	>0.9999	No
Appendix Fig 7	B, C10	Saline vs. C26 + GW	0.0013	Yes
Appendix Fig 7	B, C10	C26 vs. C26 + GW	0.0013	Yes
Appendix Fig 7	B, C12	Saline vs. C26	0.0159	Yes
Appendix Fig 7	B, C12	Saline vs. C26 + GW	0.9811	No
Appendix Fig 7	B, C12	C26 vs. C26 + GW	0.0167	Yes
Appendix Fig 7	B, C14	Saline vs. C26	0.0522	No
Appendix Fig 7	B, C14	Saline vs. C26 + GW	0.2343	No
Appendix Fig 7	B, C14	C26 vs. C26 + GW	0.0044	Yes
Appendix Fig 7	B, C16	Saline vs. C26	0.0136	Yes
Appendix Fig 7	B, C16	Saline vs. C26 + GW	0.0506	No
Appendix Fig 7	B, C16	C26 vs. C26 + GW	0.0002	Yes
Appendix Fig 7	B, C18	Saline vs. C26	0.0056	Yes
Appendix Fig 7	B, C18	Saline vs. C26 + GW	0.0053	Yes
Appendix Fig 7	B, C18	C26 vs. C26 + GW	<0.0001	Yes

Abbreviations: GW, GW274150.

Appendix Table S15. Significance p-values for Appendix Figure 8.

Figure	Panel	Comparison	p-value	Significant?
Appendix Fig 8	B, CPT1	Saline vs. GW	0.053199	No
Appendix Fig 8	B, CPT2	Saline vs. GW	0.009575	Yes
Appendix Fig 8	B, LCADH	Saline vs. GW	0.069071	No
Appendix Fig 8	B, β-oxidation	Saline vs. GW	0.02829	Yes

Abbreviations: GW, GW274150.

Appendix Table S16. Significance p-values for Appendix Figure 9.

Figure	Comparison	p-value	Significant?
Appendix Fig 9	NT vs. AMG 400μM	>0.9999	No
Appendix Fig 9	NT vs. IFNγ/TNFα	<0.0001	Yes
Appendix Fig 9	NT vs. IFNγ/TNFα + AMG 400μM	0.4721	No
Appendix Fig 9	AMG 400μM vs. IFNγ/TNFα	<0.0001	Yes
Appendix Fig 9	AMG 400μM vs. IFNγ/TNFα + AMG 400μM	0.4721	No
Appendix Fig 9	IFNγ/TNFα vs. IFNγ/TNFα + AMG 400μM	<0.0001	Yes

Abbreviations: NT, non-treated. AMG, Aminoguanidine.

Appendix Table S17. Significance p-values for Appendix Figure 10.

Figure	Panel	Comparison	p-value	Significant?
Appendix Fig 10	B	NT vs. AMG	0.2946	No
Appendix Fig 10	B	NT vs. IT	0.0062	Yes
Appendix Fig 10	B	NT vs. IT+AMG	0.0988	No
Appendix Fig 10	B	AMG vs. IT	0.0336	Yes
Appendix Fig 10	B	AMG vs. IT+AMG	0.4772	No
Appendix Fig 10	B	IT vs. IT+AMG	0.1069	No

Abbreviations: NT, non-treated. AMG, Aminoguanidine. IT, IFNγ/TNFα.

Appendix Table S18. Significance p-values for Appendix Figure 13.

Figure	Panel	Comparison	p-value	Significant?
Appendix Fig 13	B, right	NT vs. AMG 400uM	0.5327	No
Appendix Fig 13	B, right	NT vs. IFN/TNF	0.0011	Yes
Appendix Fig 13	B, right	NT vs. IFN/TNF+400 uM	0.6144	No
Appendix Fig 13	B, right	AMG 400uM vs. IFN/TNF	0.0003	Yes
Appendix Fig 13	B, right	AMG 400uM vs. IFN/TNF+400 uM	0.254	No
Appendix Fig 13	B, right	IFN/TNF vs. IFN/TNF+400 uM	0.002	Yes
Appendix Fig 13	C	NT vs. AMG 400uM	0.0283	Yes
Appendix Fig 13	C	NT vs. IFN/TNF	<0.0001	Yes
Appendix Fig 13	C	NT vs. IFN/TNF+400 uM	<0.0001	Yes
Appendix Fig 13	C	AMG 400uM vs. IFN/TNF	<0.0001	Yes
Appendix Fig 13	C	AMG 400uM vs. IFN/TNF+400 uM	<0.0001	Yes
Appendix Fig 13	C	IFN/TNF vs. IFN/TNF+400 uM	<0.0001	Yes
Appendix Fig 13	D	NT vs. AMG 400uM	0.063	No
Appendix Fig 13	D	NT vs. IFN/TNF	<0.0001	Yes
Appendix Fig 13	D	NT vs. IFN/TNF+400 uM	<0.0001	Yes
Appendix Fig 13	D	AMG 400uM vs. IFN/TNF	<0.0001	Yes
Appendix Fig 13	D	AMG 400uM vs. IFN/TNF+400 uM	<0.0001	Yes
Appendix Fig 13	D	IFN/TNF vs. IFN/TNF+400 uM	0.0014	Yes

Abbreviations: NT, non-treated. AMG, Aminoguanidine.

Appendix Table S19. Significance p-values for Appendix Figure 14.

Figure	Panel	Comparison	p-value	Significant?
Appendix Fig 14	A	NT vs. GW10	0.0784	No
Appendix Fig 14	A	NT vs. GW100	0.0033	Yes
Appendix Fig 14	A	NT vs. IT	0.4675	No
Appendix Fig 14	A	NT vs. IT+GW10	0.3609	No
Appendix Fig 14	A	NT vs. IT+GW100	0.1847	No
Appendix Fig 14	A	GW10 vs. GW100	0.1469	No
Appendix Fig 14	A	GW10 vs. IT	0.2758	No
Appendix Fig 14	A	GW10 vs. IT+GW10	0.3653	No
Appendix Fig 14	A	GW10 vs. IT+GW100	0.6321	No
Appendix Fig 14	A	GW100 vs. IT	0.0166	Yes
Appendix Fig 14	A	GW100 vs. IT+GW10	0.025	Yes
Appendix Fig 14	A	GW100 vs. IT+GW100	0.0605	No
Appendix Fig 14	A	IT vs. IT+GW10	0.8473	No
Appendix Fig 14	A	IT vs. IT+GW100	0.5322	No
Appendix Fig 14	A	IT+GW10 vs. IT+GW100	0.664	No
Appendix Fig 14	B	NT vs. GW10	0.8279	No
Appendix Fig 14	B	NT vs. GW100	0.7165	No
Appendix Fig 14	B	NT vs. IT	0.0426	Yes
Appendix Fig 14	B	NT vs. IT+GW10	0.0149	Yes
Appendix Fig 14	B	NT vs. IT+GW100	0.0351	Yes
Appendix Fig 14	B	GW10 vs. GW100	0.8837	No
Appendix Fig 14	B	GW10 vs. IT	0.0655	No
Appendix Fig 14	B	GW10 vs. IT+GW10	0.0236	Yes
Appendix Fig 14	B	GW10 vs. IT+GW100	0.0543	No
Appendix Fig 14	B	GW100 vs. IT	0.0865	No
Appendix Fig 14	B	GW100 vs. IT+GW10	0.032	Yes
Appendix Fig 14	B	GW100 vs. IT+GW100	0.0721	No
Appendix Fig 14	B	IT vs. IT+GW10	0.6156	No
Appendix Fig 14	B	IT vs. IT+GW100	0.9233	No
Appendix Fig 14	B	IT+GW10 vs. IT+GW100	0.6842	No
Appendix Fig 14	C	NT vs. GW10	0.2751	No
Appendix Fig 14	C	NT vs. GW100	0.3148	No
Appendix Fig 14	C	NT vs. IT	0.0174	Yes
Appendix Fig 14	C	NT vs. IT+GW10	0.0108	Yes
Appendix Fig 14	C	NT vs. IT+GW100	0.0218	Yes
Appendix Fig 14	C	GW10 vs. GW100	0.9279	No
Appendix Fig 14	C	GW10 vs. IT	0.1527	No
Appendix Fig 14	C	GW10 vs. IT+GW10	0.1028	No
Appendix Fig 14	C	GW10 vs. IT+GW100	0.1831	No
Appendix Fig 14	C	GW100 vs. IT	0.1304	No
Appendix Fig 14	C	GW100 vs. IT+GW10	0.087	No

Appendix Fig 14	C	GW100 vs. IT+GW100	0.1571	No
Appendix Fig 14	C	IT vs. IT+GW10	0.8242	No
Appendix Fig 14	C	IT vs. IT+GW100	0.9147	No
Appendix Fig 14	C	IT+GW10 vs. IT+GW100	0.7422	No

Abbreviations: NT, non-treated. GW, GW274150. IT, IFNγ/TNFα.
