# Supplementary material for: Pharmacological or genetic inhibition of iNOS prevents cachexia‐mediated muscle wasting and its associated metabolism defects
Source: EMBO Mol Med. 2021 Jun 7;13(7):e13591. doi: 10.15252/emmm.202013591 (PMC8261493; doi:10.15252/emmm.202013591)
Supplement: Supplementary file 2 — Expanded View Figures PDF [file EMMM-13-e13591-s002.pdf]

Expanded View Figures

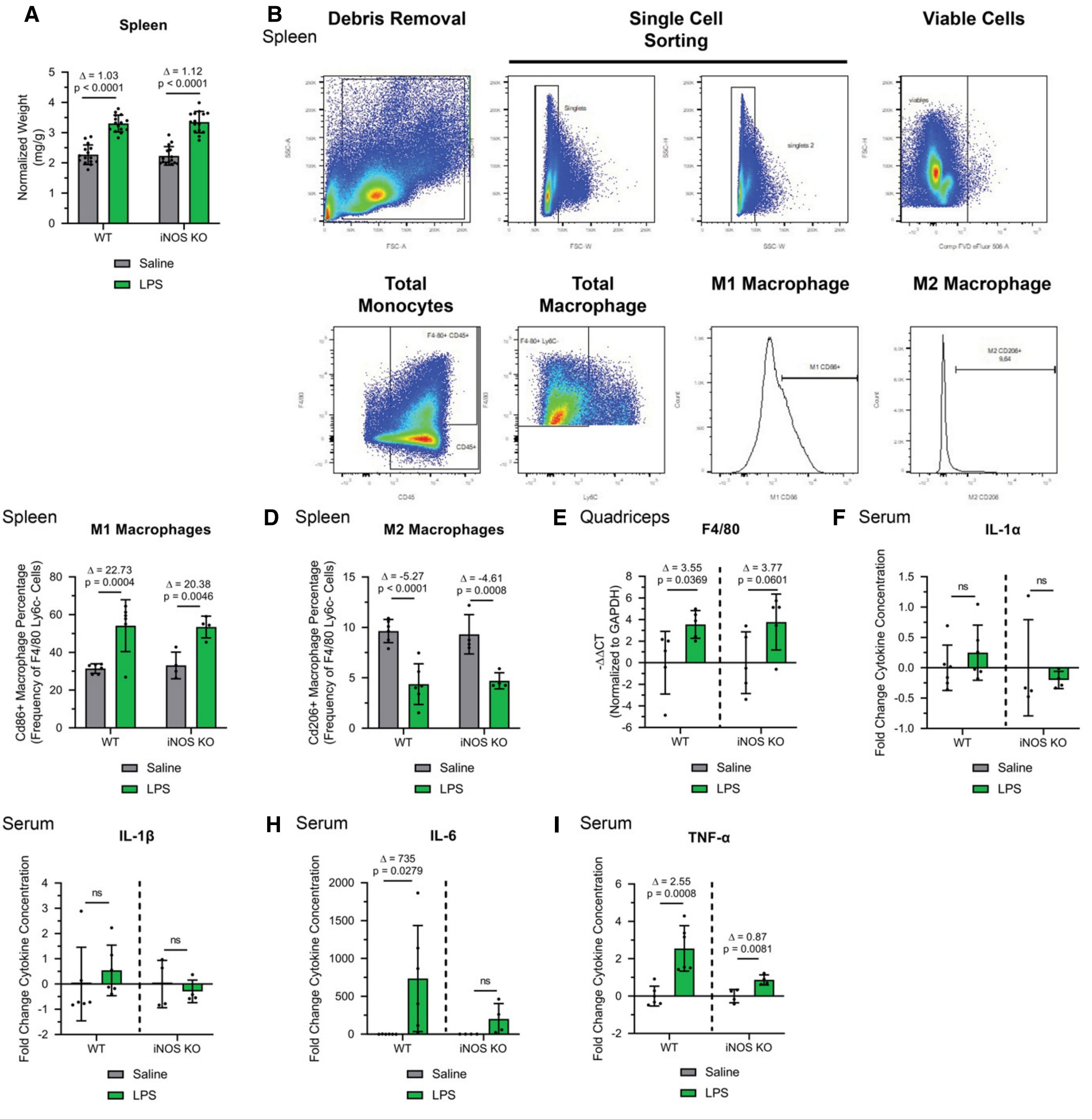

Figure EV1

Figure EV1. Characterization of the LPS-induced immune response in WT and iNOS KO mice.

Male C57BL/6 wild-type (WT) and iNOS knockout (KO) mice were intraperitoneally injected with 1 mg kg^{-1} LPS or an equivalent volume of carrier solution. Control WT, control KO, and LPS-treated KO cohorts were pair-fed (PF) to the WT LPS-treated cohorts. After 18 h, mice were euthanized, and tissue samples were analyzed.

A Spleen weight normalized to initial body weight (n = 15).

B Gating strategy for flow cytometry analysis of spleen M1 and M2 macrophages.

C Percentage of $CD86^+$ M1 splenic macrophages relative to the total F4/80+Ly6C- macrophage population (WT saline n = 6, WT LPS n = 6, iNOS KO saline n = 4, and iNOS KO LPS n = 4).

D Percentage of $CD206^+$ M2 splenic macrophages relative to the total F4/80+Ly6C- macrophage population (WT saline n = 6, WT LPS n = 6, iNOS KO saline n = 4, and iNOS KO LPS n = 4).

E *Quadriceps* from saline or LPS-treated, WT or iNOS KO mice were isolated and used for RT–qPCR analysis for F4/80 mRNA expression. The $-\Delta\Delta$ CT was plotted relative to the saline conditions of each background and normalized to GAPDH mRNA levels (n = 5).

F–I Serum from saline or LPS-treated, WT or iNOS KO mice was collected. Fold change in IL-1α (F), IL-1β (G), IL-6 (H), and TNF-α (I) levels in LPS-treated mice was plotted relative to saline condition (WT saline n = 6, WT LPS n = 6, iNOS KO saline n = 4, and iNOS KO LPS n = 4).

Data information: Individual data points represent values from individual mice. Error bars represent the standard deviation (SD) of the mean. (A, C, D) For statistical comparisons, Δ indicates the difference in mean values and P-values were calculated with an ANOVA followed by Fisher's LSD test. (E-I) Statistical comparisons were made between saline-treated controls and LPS-treated mice of same genotype. Plotted concentration data were relativized to saline controls of corresponding genotype. Δ indicates the difference in mean values, and P-values were calculated with Student's t-test.

 © 2021 The Authors

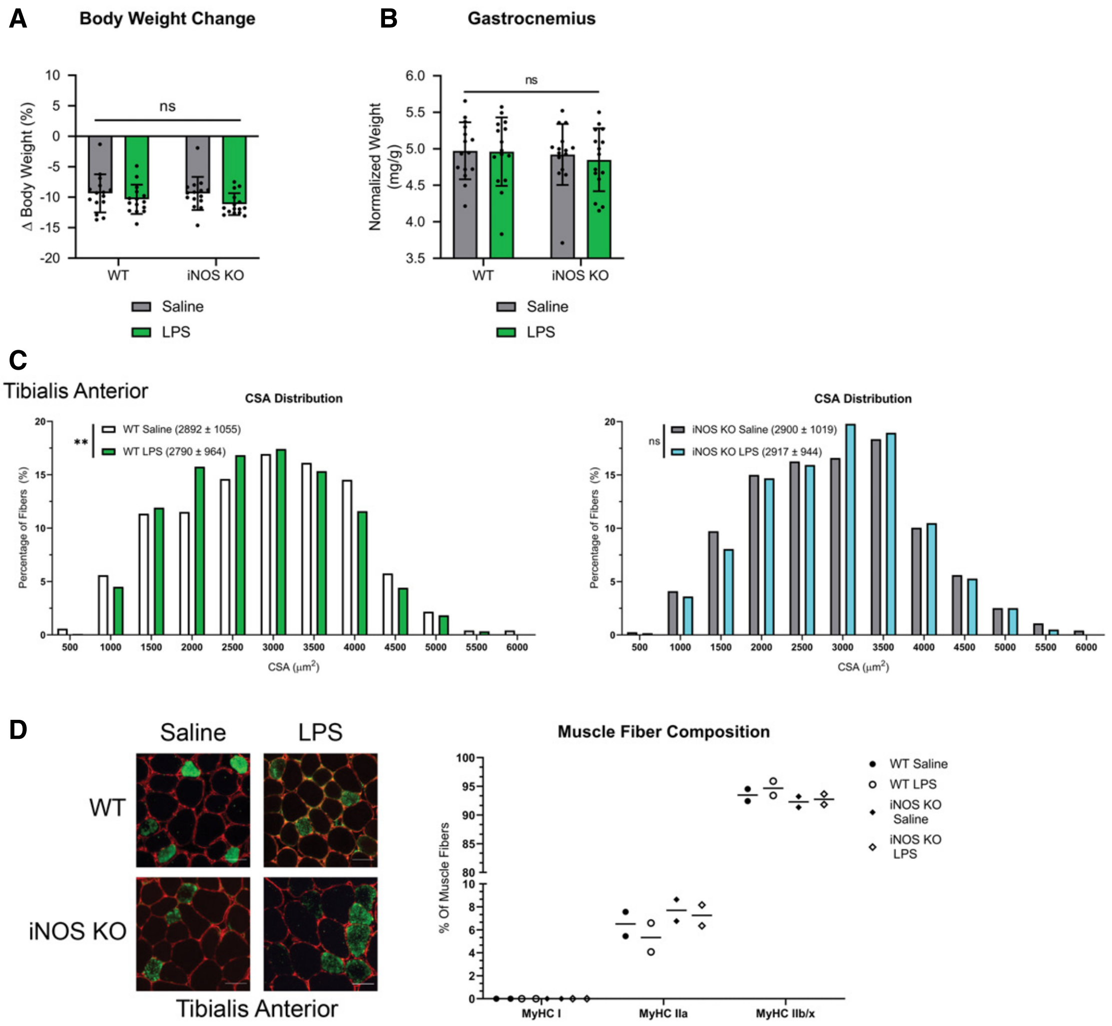

Figure EV2. Characterization of the LPS-induced effects on muscle integrity/composition in WT and iNOS KO mice.

Male C57BL/6 wild-type (WT) and iNOS knockout (KO) mice were intraperitoneally injected with 1 mg kg^{-1} LPS or an equivalent volume of carrier solution. Control WT, control KO, and LPS-treated KO cohorts were pair-fed (PF) to the WT LPS-treated cohorts. After 18 h, mice were euthanized, and tissue samples were analyzed.

A Percent body weight change from time of injection to endpoint of experiment. (n = 15).

B *Gastrocnemius* weight normalized to initial body weight (n = 15).

C Frequency histogram showing the distribution of muscle fiber CSA in the *tibialis anterior* muscles from (*left*) WT control and (*right*) iNOS KO mice (n = 4). A total of 300 fibers per muscle were used for the CSA analysis. Statistical comparisons, mean, and standard deviation of the mean are shown in the histogram legend.

D (*left*) Photomicrographs of immunofluorescence images of *tibialis anterior* muscle sections from WT control and iNOS KO mice injected with or without LPS. Sections were stained against MyHC I (Blue), MyHC IIa (Green), and laminin (Red). Unstained fibers were classified as MyHC IIb/x. Scale bars = 50 μm. (*right*) Percentage of type I, type IIa, and type IIb/IIx fibers in the whole *tibialis anterior* muscles from control and iNOS KO mice (n = 2).

Data information: Individual data points represent values from individual mice. Error bars represent the standard deviation (SD) of the mean. (C) P-values were calculated with a Kolmogorov–Smirnov test (**P < 0.01). Non-statistically significant comparisons (P > 0.05) are indicated as non-significant (ns).

© 2021 The Authors

Figure EV3. Effect of GW on the immune response as well as muscle integrity/composition in C26-tumor-bearing mice.

Male BALB/C mice were injected subcutaneously with C26 cells (1.25×10^6 cells) or an equivalent volume of saline. After 5 days, and everyday thereafter, saline- and C26-injected mice were treated with or without GW (5 mg kg^{-1}). After 16 days, mice were euthanized and tissue samples were analyzed.

A iNOS and tubulin (loading control) protein levels in the *gastrocnemius* muscle were assessed by Western blot ($n = 3$).

B Spleen weight normalized to initial body weight.

C *Quadriceps* from control and C26 mice treated with or without GW were isolated and used for RT–qPCR analysis for F4/80 mRNA expression. The $-\Delta\Delta$ CT is plotted relative to the saline condition and normalized to GAPDH mRNA levels ($n = 5$).

D–G Serum from saline and C26-tumor-bearing mice treated with or without GW was collected. Fold change in IL-1α (D), IL-1β (E), IL-6 (F), and TNF-α (G, $n = 4$ for C26 + GW) levels was plotted relative to saline condition.

E Inguinal fat pad weight normalized to initial body weight.

F Frequency histogram showing the distribution of muscle fiber CSA in the *gastrocnemius* muscles from control and C26 mice treated with or without GW (saline $n = 4$, C26 $n = 4$, GW 5 mg/kg $n = 3$, and C26 GW 5 mg/kg $n = 4$). A total of 600–700 fibers per muscle were used for the CSA analysis. Statistical comparisons, mean, and standard deviation of the mean are shown in the histogram legend.

G (*left*) Photomicrographs of immunofluorescence images of *gastrocnemius* muscle sections from control and C26 mice treated with or without GW. Sections were stained against MyHC I (Blue), MyHC IIa (Green), and laminin (Red). Unstained fibers were classified as MyHC IIb/x. Scale bars = 50 μm. (*right*) Percentage of type I, type IIa, and type IIb/IIx fibers in the whole gastrocnemius muscles from control and C26 mice treated with or without GW ($n = 2$).

Data information: Individual data points represent values from individual mice, with a total of six mice per cohort ($n = 6$) unless stated otherwise. Error bars represent the standard deviation (SD) of the mean. (B–H) For statistical comparisons, Δ indicates the difference in mean values and *P*-values were calculated with an ANOVA followed by Fisher's LSD test. (I) *P*-values were calculated with a Kolmogorov–Smirnov test (***$P < 0.001$).

Source data are available online for this figure.

© 2021 The Authors

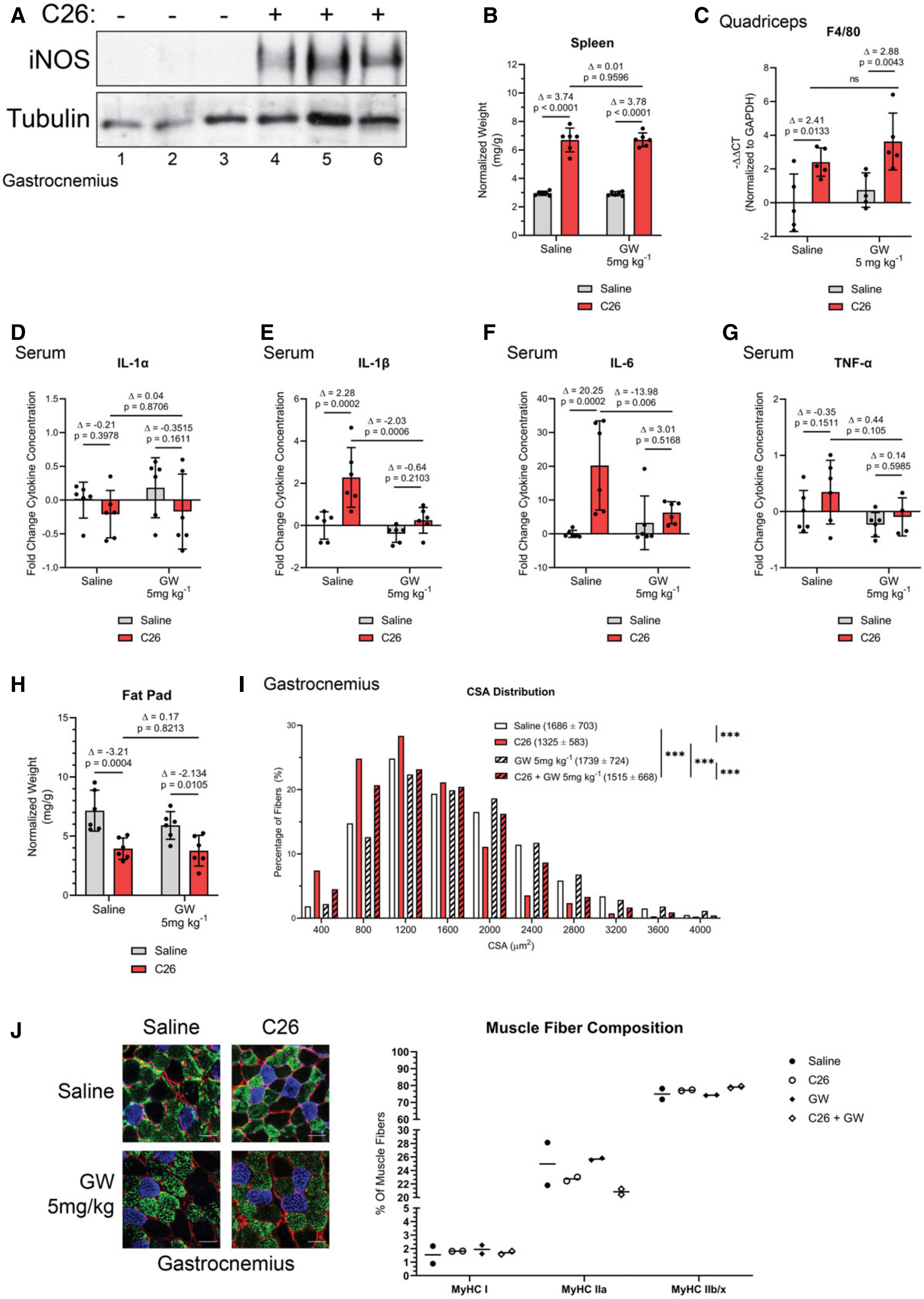
A
C26: - - - + + +
iNOS
Tubulin
1 2 3 4 5 6
Gastrocnemius
B
Spleen
Normalized Weight (mg/g)
Δ = 3.74 p < 0.0001
Δ = 3.78 p < 0.0001
Δ = 0.01 p = 0.9596
Saline
GW 5mg kg-1
C Quadriceps
F4/80
-ΔΔCT (Normalized to GAPDH)
Δ = 2.41 p = 0.0133
Δ = 2.88 p = 0.0043
ns
D Serum
IL-1α
E Serum
IL-1β
F Serum
IL-6
G Serum
TNF-α
Fold Change Cytokine Concentration
H
Fat Pad
I Gastrocnemius
CSA Distribution
Saline (1686 ± 703)
C26 (1325 ± 583)
GW 5mg kg-1 (1739 ± 724)
C26 + GW 5mg kg-1 (1515 ± 668)
Percentage of Fibers (%)
CSA (μm2)
J
Saline C26
Saline
GW 5mg/kg
Gastrocnemius
Muscle Fiber Composition
% Of Muscle Fibers
MyHC I
MyHC IIa
MyHC IIb/x
Saline
C26
GW
C26 + GW

Figure EV3.

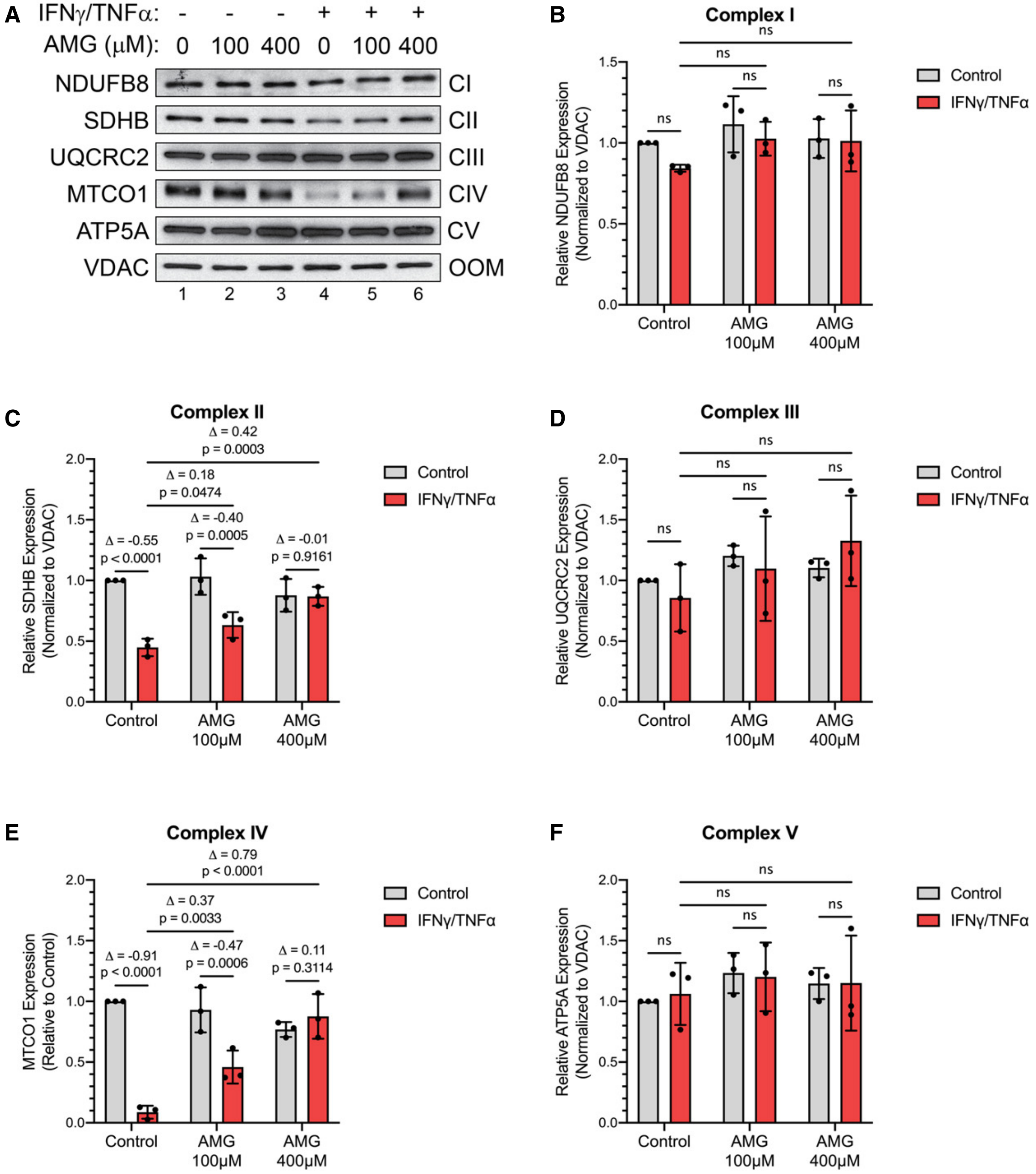

Figure EV4. Cytokine-mediated loss of complex II and IV integrity is reversed with aminoguanidine.

C2C12 myotubes were treated with or without IFNγ (100 U/ml) and TNF-α (20 ng/ml) and the indicated doses of aminoguanidine (AMG). Protein content was extracted 24 h after treatment.

A Western blot analysis for ETC protein complex subunits.

B–F Quantification of complex subunits normalized to VDAC (outer mitochondrial membrane; OMM) and relative to untreated control. (B) NDUFB8 (complex I; CI), (C) SDHB (complex II; CII), (D) UQCRC2 (complex III; CIII), (E) MTCO1 (complex IV; CIV), and (F) ATP5A (complex V; CV).

Data information: Individual data points are from three independent experimental replicates ($n = 3$). Error bars represent the standard deviation (SD) of the mean. For statistical comparisons, Δ indicates the difference in mean values and P-values were calculated with an ANOVA followed by Fisher's LSD test. Non-statistically significant comparisons ($P > 0.05$) are indicated as non-significant (ns).

Source data are available online for this figure.

© 2021 The Authors
