# Supplementary material for: Pharmacological or genetic inhibition of iNOS prevents cachexia‐mediated muscle wasting and its associated metabolism defects
Source: EMBO Mol Med. 2021 Jun 7;13(7):e13591. doi: 10.15252/emmm.202013591 (PMC8261493; doi:10.15252/emmm.202013591)

Figure EV3

Uncropped Blots for Figure EV3a

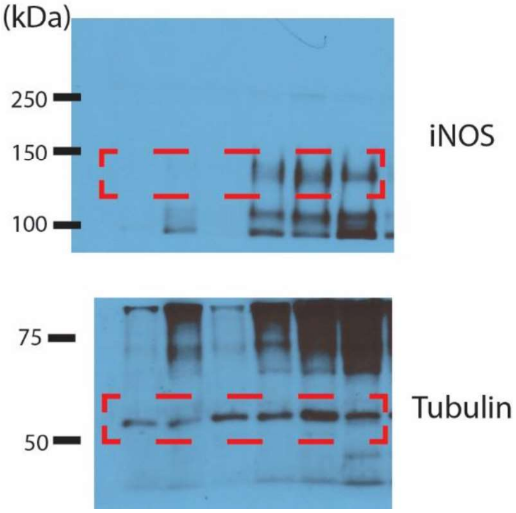

Figure EV4

Uncropped Blots for Figure EV4

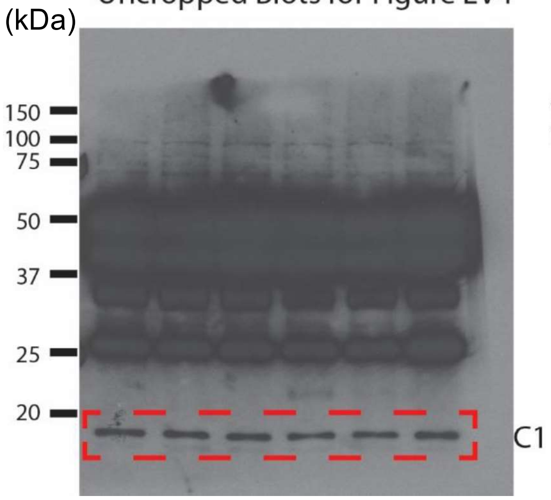

Uncropped Blots for Figure EV4

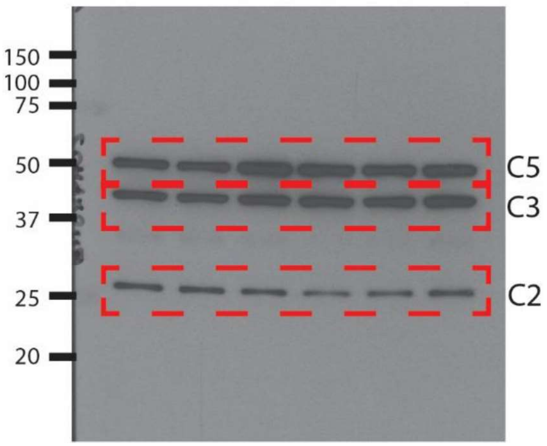

Uncropped Blots for Figure EV4

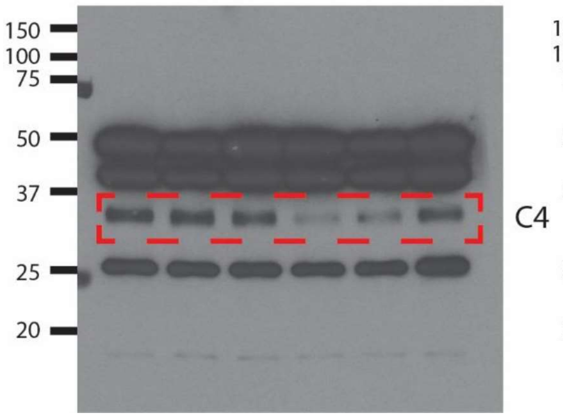

Uncropped Blots for Figure EV4

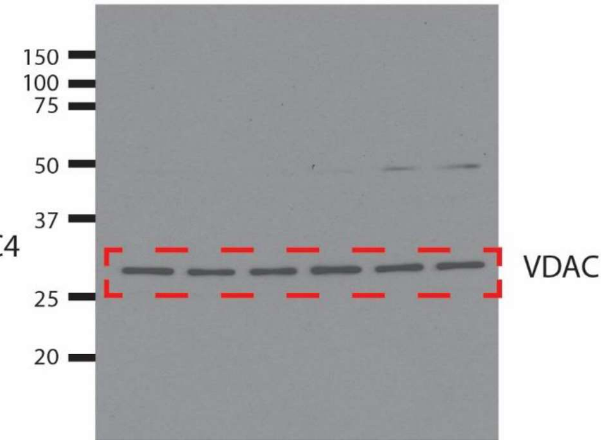

Appendix Figure 10

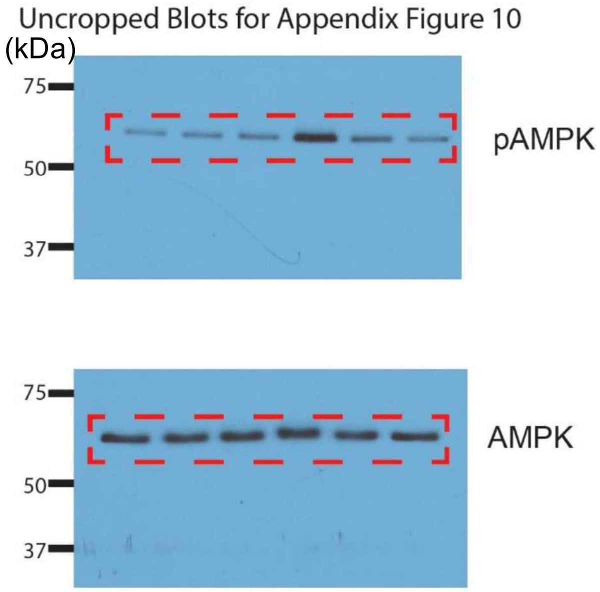

Supplement: Supplementary file 9 — Source Data for Expanded View/Appendix [file EMMM-13-e13591-s015.zip › emmm202013591-sup-0015-SDataEV/emmm202013591-sup-0015-SDataEV.pdf]
