# Supplementary figures and images for: Pharmacological or genetic inhibition of iNOS prevents cachexia‐mediated muscle wasting and its associated metabolism defects
Source: EMBO Mol Med. 2021 Jun 7;13(7):e13591. doi: 10.15252/emmm.202013591 (PMC8261493; doi:10.15252/emmm.202013591)

**Source Data file:**

**Figure 1**

Uncropped Blots for Figure 1A

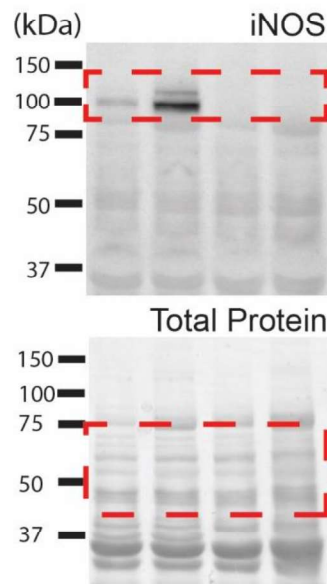

Uncropped Blots for Figure 1B

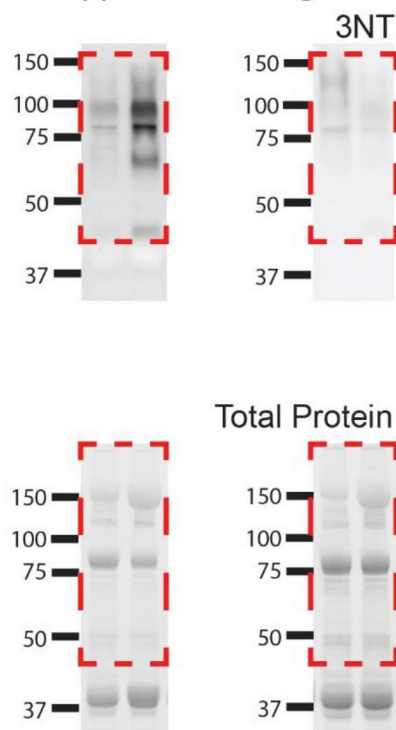

Supplement: Supplementary file 11 — Source Data for Figure 1 [file EMMM-13-e13591-s008.pdf]

Figure 2

Uncropped Blots for Figure 2C

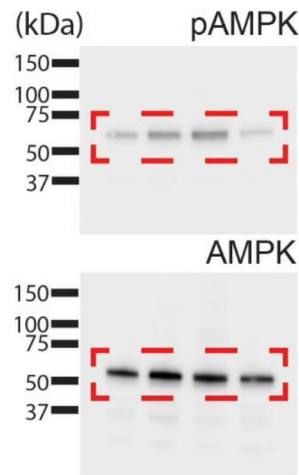

Supplement: Supplementary file 12 — Source Data for Figure 2 [file EMMM-13-e13591-s014.pdf]

**Figure 3**

Uncropped Blots for Figure 3B

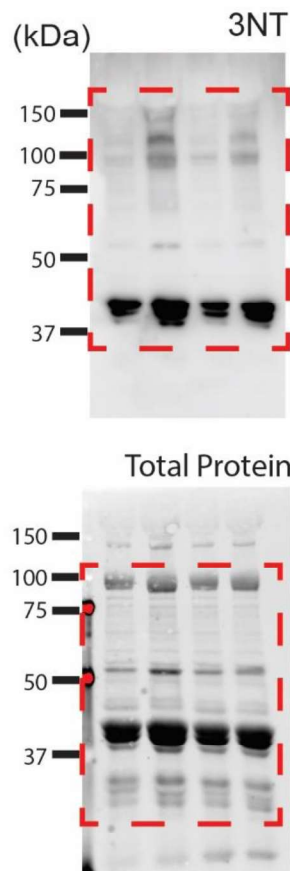

Supplement: Supplementary file 13 — Source Data for Figure 3 [file EMMM-13-e13591-s001.pdf]

**Figure 4**

Uncropped Blots for Figure 4C

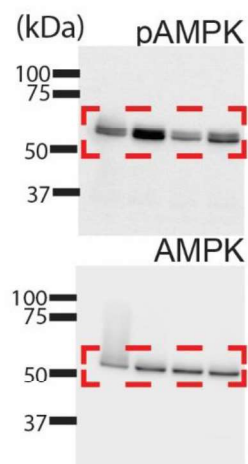

Supplement: Supplementary file 14 — Source Data for Figure 4 [file EMMM-13-e13591-s016.pdf]

**Figure 5**

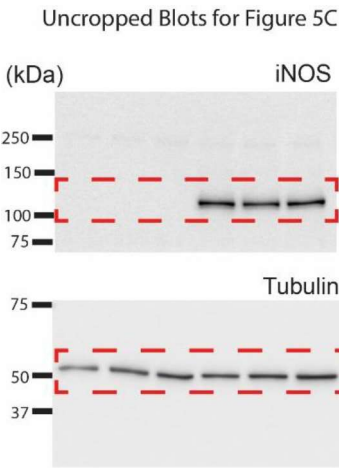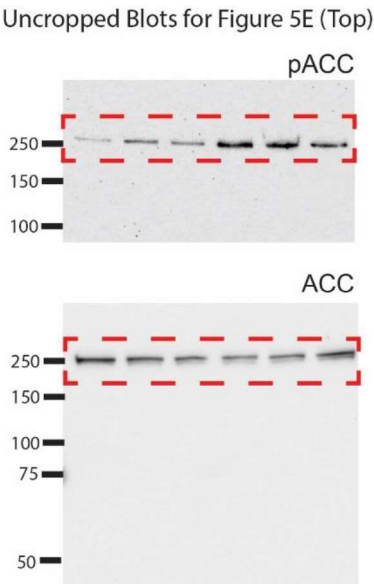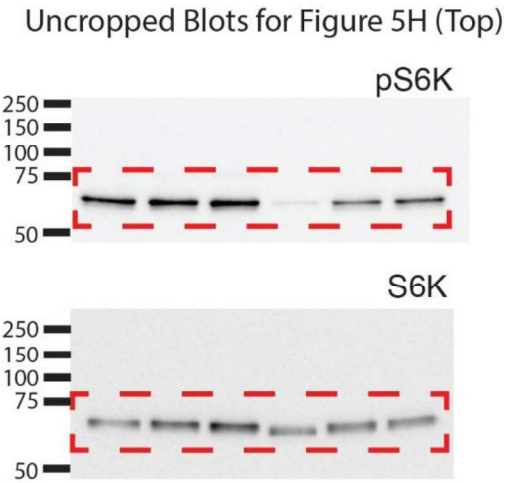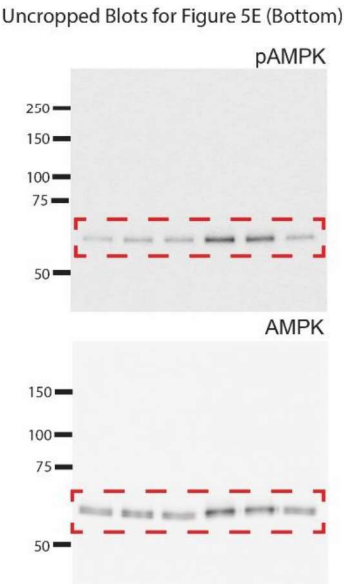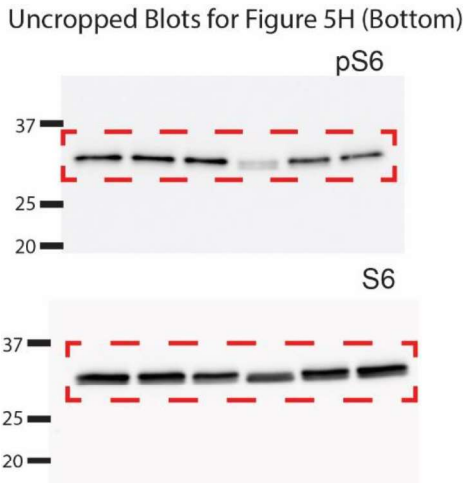

Supplement: Supplementary file 15 — Source Data for Figure 5 [file EMMM-13-e13591-s012.pdf]
