# Supplementary material for: Pharmacological or genetic inhibition of iNOS prevents cachexia‐mediated muscle wasting and its associated metabolism defects
Source: EMBO Mol Med. 2021 Jun 7;13(7):e13591. doi: 10.15252/emmm.202013591 (PMC8261493; doi:10.15252/emmm.202013591)
Supplement: Supplementary file 16 — Source Data for Figure 7 [file EMMM-13-e13591-s013.pdf]

Figure 7

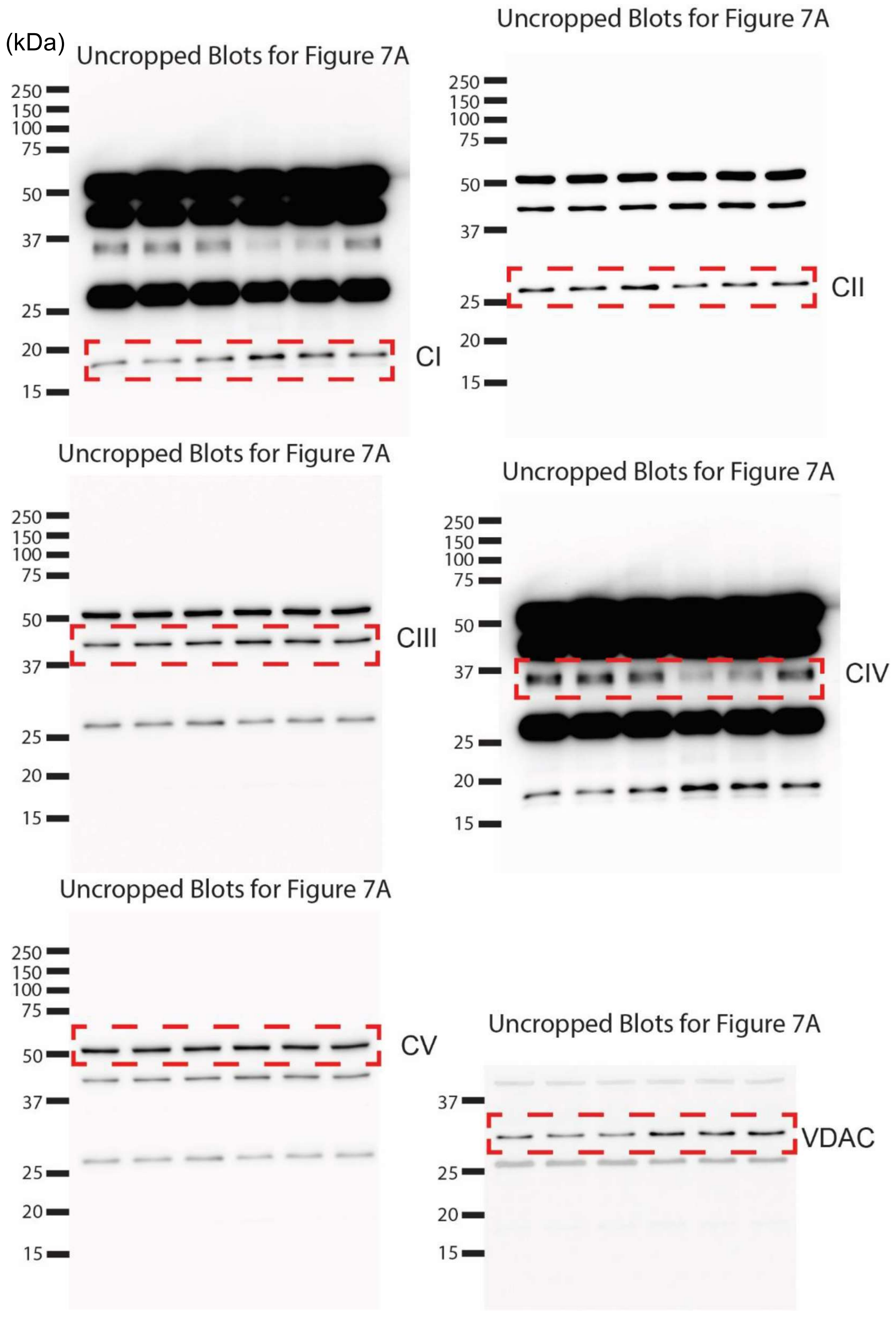
(kDa)
Uncropped Blots for Figure 7A
250
150
100
75
50
37
25
20
15
CI
Uncropped Blots for Figure 7A
250
150
100
75
50
37
25
20
15
CII
Uncropped Blots for Figure 7A
250
150
100
75
50
37
25
20
15
CIII
Uncropped Blots for Figure 7A
250
150
100
75
50
37
25
20
15
CIV
Uncropped Blots for Figure 7A
250
150
100
75
50
37
25
20
15
CV
Uncropped Blots for Figure 7A
37
25
20
15
VDAC
